# Transfer learning of hyperparameters for fast construction of anisotropic GPR models: design and application to the machine-learned force field FFLUX†

**DOI:** 10.1039/d4cp01862a

**Published:** 2024-09-03

**Authors:** Bienfait K. Isamura, Paul L. A. Popelier

**Affiliations:** a Department of Chemistry, The University of Manchester Manchester M13 9PL UK paul.popelier@manchester.ac.uk

## Abstract

The polarisable machine-learned force field FFLUX requires pre-trained anisotropic Gaussian process regression (GPR) models of atomic energies and multipole moments to propagate unbiased molecular dynamics simulations. The outcome of FFLUX simulations is highly dependent on the predictive accuracy of the underlying models whose training entails determining the optimal set of model hyperparameters. Unfortunately, traditional direct learning (DL) procedures do not scale well on this task, especially when the hyperparameter search is initiated from a (set of) random guess solution(s). Additionally, the complexity of the hyperparameter space (HS) increases with the number of geometrical input features, at least for anisotropic kernels, making the optimization of hyperparameters even more challenging. In this study, we propose a transfer learning (TL) protocol that accelerates the training process of anisotropic GPR models by facilitating access to promising regions of the HS. The protocol is based on a seeding–relaxation mechanism in which an excellent guess solution is identified by rapidly building one or several small source models over a subset of the target training set before readjusting the previous guess over the entire set. We demonstrate the performance of this protocol by building and assessing the performance of DL and TL models of atomic energies and charges in various conformations of benzene, ethanol, formic acid dimer and the drug fomepizole. Our experiments suggest that TL models can be built one order of magnitude faster while preserving the quality of their DL analogs. Most importantly, when deployed in FFLUX simulations, TL models compete with or even outperform their DL analogs when it comes to performing FFLUX geometry optimization and computing harmonic vibrational modes.

## Abstract 2

This work introduces a transfer learning (TL) protocol that accelerates the training of anisotropic Gaussian process regression models while preserving their excellent predictive capability.

## Graphical abstract

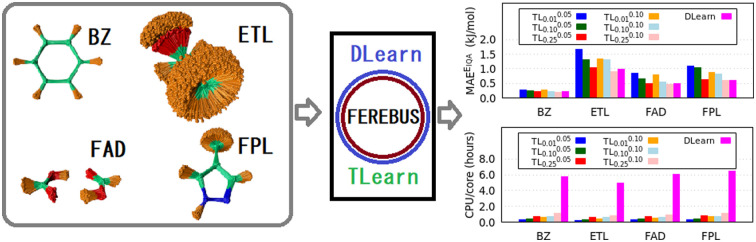

## 1. Introduction

The artificial intelligence revolution has recently caused major paradigm shifts in many research fields resulting in the promotion of various unconventional data-driven approaches. In computational chemistry, the integration of traditional simulation techniques with machine learning has given rise to a new class of unconventional force fields known as machine-learned potentials (MLPs).^1–3^ These next-generation force fields find their motivation in the necessity to circumvent the exorbitant cost of on-the-fly electronic structure calculations.

In essence, MLPs aim to reach the accuracy of high-level quantum mechanics approaches at a cost comparable to that of classic force fields. They owe their current success to the predictive capability and efficiency of the underlying machine learning (ML) models, which are trained on high-quality electronic energies and/or forces. Once trained, these models can be deployed in simulations to rapidly evaluate the same properties on previously unseen compounds. The development of various interfaces with existing computational packages has facilitated the deployment of MLPs in simulations,^4^ enabling these packages to accomplish fast and accurate ML-aided geometry optimizations and MD simulations.^5–7^

FFLUX is a polarisable MLP that relies on pre-trained Gaussian process regression (GPR) models of atomic energies and multipole moments to action molecular dynamics (MD) simulations.^8^ This next-generation force field has been successfully utilized to investigate the bulk properties of liquid water^9^ and the polymorphism of formamide,^10^ to cite only these examples. The outcome of FFLUX simulations depends heavily on the predictive capability of the underlying models, which is in turn dictated by the choice of model hyperparameters. Regrettably, optimal hyperparameters are never known in advance. Instead, they must be determined by navigating the landscape of a given loss function. Because tiny changes in the hyperparameters’ values can cause disproportional fluctuations in the quality of a model, it is important that optimal hyperparameters be accurately determined. Unfortunately, the exercise of fine-tuning GPR model hyperparameters does not scale well with the size of the training set, namely 𝒪(*N*^3^) in terms of computational complexity and 𝒪(*N*^2^) for memory. Even more annoying is the complexity of the hyperparameter space, which usually blows up with the number of input features, at least for anisotropic kernels, making it even more challenging to locate optimal kernel parameters within a reasonable amount of time.

The unfavorable scaling of GPs is the main bottleneck discouraging their application to large datasets. Several solutions have been proposed to address this problem. For brevity, we will only mention the two most conceptually appealing solutions. The first solution consists of applying so-called reduced-rank approximations.^11^ These sparse GPs have been proven to considerably speed up the training process of GPR models while reducing the memory requirement. These schemes attempt to avoid any explicit manipulation (especially inversion) of the full covariance matrix but work on projections in different subspaces. Popular sparse GP algorithms exhibit 𝒪(*N*^2^*N*) computational complexity and 𝒪(*MN*) storage demand, with *M* (*M* < *N*) being the number of active points.^12^ Despite this outstanding improvement, these schemes often fail to preserve the desired predictive accuracy of full GP inference. This is because the selection of the so-called “inducing points, support points, pseudo-points or even active points is more challenging than it may seem and when inappropriately done, might have unexpected and usually disagreeable repercussions on the sparsity, numerical stability, and predictive accuracy of sparse GPs. Furthermore, as long as the selection of active/pseudo-points is coupled with the optimization of hyperparameters, there are more variables to fit, which (unless carefully handled) increases the risk of overfitting as recognized in the case of sparse spectrum GPR.^13^

The second solution exploits the nature of the solver used to invert the covariance matrix. Indeed, there is empirical proof that GPR models can be trained more effectively if an iterative solver, like a preconditioned conjugate gradient, were used in place of the usual Cholesky decomposition, which is a direct solver.^14,15^ It is true that iterative solvers not only eliminate the need for storing the entire covariance matrix but also reduce the complexity from 𝒪(*N*^3^) to 𝒪(*N*^2^). However, since their convergence is never guaranteed, even when boosted by tailored preconditioners, it is not always clear how much the hyperparameters, the regression weights, and subsequently the quality of the final model have been compromised. Therefore, although elegant and recommended for extremely large datasets, both sparse GPs and iterative solvers only offer a tangible advantage when memory allocation is a concern. Otherwise, to preserve the outstanding and desired predictive capability of exact GP inference on (relatively) big yet manageable datasets, alternative strategies for fast training of full GPR models employing direct solvers must be promoted.^16,17^

Assuming the covariance matrix can fit in memory, it is right to think that GPR models can be built more efficiently by minimizing the number of times the full covariance matrix must be inverted when optimizing model GPR hyperparameters. One way to achieve this is to ensure that the tuning of a target model is started from an excellent, yet easily accessible, set of hyperparameters. Having an excellent guess solution, as opposed to random initialization, is expected to facilitate the location of promising areas of the hyperparameter space (HS) and, as a result, to naturally reduce the number of iterations required to locate a reasonable estimation of the optimal hyperparameters. However, since each HS is unique, finding a good starting set of hyperparameters is never trivial, and may become impossible if one has to set it up manually. Here we propose a protocol that systematically solves the problem, based on the concept of “transfer of knowledge (hyperparameters)” or transfer learning (TL).

The current proof-of-concept study aims (i) to demonstrate the transferability of hyperparameters in anisotropic GP regression, and (ii) to document the balance between the predictive accuracy and building cost of atomic GPR models trained *via* direct and transfer learning of hyperparameters. The protocol presented in Section 2.4.1 has been implemented in our in-house program FEREBUS.^18–20^ The latter program is a GPR engine written in free format modern Fortran, and accelerated *via* Open Multi-Processing. We claim the universality of this TL protocol as it does not impose any specific requirement in terms of datasets. The only design choices are that (i) the covariance matrix fits in memory, (ii) each GPR model is trained to reproduce a unique target property (single-objective GPR), and (iii) the chosen kernel is anisotropic and defined in terms of automatic relevance determination. The third design choice can be justified by evoking the well-known higher flexibility and superior predictive capability of anisotropic GPR models as compared to their isotropic analogs.^21^

The protocol proposed here follows a two-phase seeding–relaxation mechanism in which an excellent guess solution is located by training one (or possibly several) source model(s) over a subset of the (target) training set, before relaxing that guess for a few iterations on the entire training set. The protocol has been tested by building and deploying both direct and TL models in FFLUX simulations. These models were trained on the electronic energies and charges of topological quantum atoms in various conformations of benzene (BZ), formic acid dimer (FAD), ethanol (ETL), and fomepizole (FPL) (4-methylpyrazole). As in previous studies from our group, we rely on quantum chemical topology (QCT)^22^ tools to realize an exhaustive real-space partitioning of molecular properties into atomic/local contributions. However, unlike the usual assessment of our GPR models based on atom-wise predictions,^23,24^ we focus here on the ability of these atomic GPR models to reconstruct molecular energies and charges. This allows us to obtain an overall appreciation of the quality of all atomic models without having to explicitly look at each of them.

We show (i) that TL of hyperparameters accelerates the training process of anisotropic GPR models while preserving the predictive accuracy of direct models, (ii) that frozen-seed (FS) TL models, *i.e.* TL modes whose guess solution (seed) was not relaxed, can suffer from sub-optimality when the source dataset is small and non-representative of the target dataset and (iii) that TL models exhibit competitive performance to their DL analogs when deployed in FFLUX simulations.

The remainder of this paper is organized as follows: in Section 2, we cover the theory this work is built on. Computational details, including dataset generation, model construction, optimization settings, and FFLUX simulation details are presented in Section 3. Section 4 is devoted to presenting and discussing our results, focusing on the learning capability of DL models and the comparison between DL and TL models. Finally, we reiterate the main findings in the conclusion.

## 2. Theory

### 2.1. FFLUX: a polarisable QCT-based force field

The logical premise underlying the architecture of most MLPs is that the total energy *E* of a molecular system can be decomposed into a sum of local contributions *E*_*i*_, *i.e.*, 
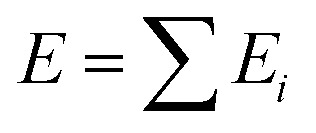
. This partitioning procedure is either automated within the ML architecture^25,26^ or carefully performed before training.^8^ In the first case, one obtains a collection of “sites” energies that might fluctuate in unexpected ways depending on the learning architecture. Unlike pre-calculated, physically justified atomic energies, these quantities may not reflect any well-defined physical quantity. The second option is the choice of the polarisable force field FFLUX. The latter relies on atomic GPR models of justifiable atomic energies and multipole moments.

The reference (“exact”) atomic energies and multipole moments that our GPR models are trained on are determined using a combination of two QCT methods: the quantum theory of atoms in molecules (QTAIM)^27^ and the interacting quantum atoms (IQA)^28^ energy decomposition method.

On the one hand, QTAIM allows molecules and clusters to be compartmented into non-overlapping regions/basins known as topological quantum atoms. Each such quantum atom *Ω* is surrounded by an interatomic surface *S*(*Ω*) characterized by a zero flux of the gradient vector field of the electron density ∇*ρ*(***r***).

1 ∇*ρ*(***r***)·***n***(***r***) = 0 for all points on *S*

where ***r*** ∈ *S*(***r***) and ***n***(***r***) is a unit vector perpendicular to *S*(*Ω*) at ***r***.

On the other hand, the IQA^29^ methodology exploits the first and second-order reduced density matrices to exhaustively decompose the total energy of a molecular system into atomic contributions *E*^A^_IQA_. Each atomic IQA energy encloses an intra-atomic (*E*^A^_intra_) and an interatomic (*E*^AB^_intra_) component (eqn (2)). The latter components can be further partitioned into finer contributions as shown in eqn (3) and (4).2
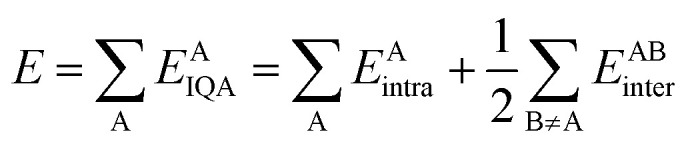

3 *E*^A^_intra_ = *T*^*A*^ + *V*^A^_ne_ + *V*^A^_ee_

4 *V*^AB^_inter_ = *V*^AB^_nn_ + *V*^AB^_ne_ +*V*^AB^_en_ + *V*^AB^_ee_

where the left-most sum in eqn (2) runs over all topological atoms, while the right-most sum runs over all the other atomic basins B. The subscripts n and e in eqn (3) and (4) stand respectively for nuclear and electronic interactions.

The fact that *V*^AB^_ee_ can be split into a Coulombic (*V*^AB^_cout_) and an exchange–correlation (*V*^AB^_xc_) energy term makes it possible to rewrite *V*^AB^_inter_ in a more compact way as the sum of a purely classical (*V*^AB^_cl_) and an exchange–correlation term (*V*^AB^_xc_).

5 *V*^AB^_inter_ = *V*^AB^_cl_ + *V*^AB^_xc_

Note that atomic multipole moments (*Q*_*lm*_) are obtained *via* a Taylor expansion of *V*^AB^_cl_.

Our in-house MD simulator DL-FFLUX^8^ utilizes flexible multipole moments (up to *Q*_*4m*_) to describe long-range electrostatic interactions, while short-range interactions are captured within *E*_IQA_. Only *E*_IQA_ models are required to action FFLUX simulations of single molecules in the gas phase such as the ones reported in this study.

### 2.2. Gaussian process regression

Gaussian process (GP) regression is a non-parametric interpolation technique that provides both accurate predictions and reliable estimates of the uncertainty associated with each prediction. The approach can be derived from Bayesian statistics building on the concept of “Gaussian processes”. For the sake of brevity, we recommend to interested readers the seminal book by Rasmussen and Williams.^11^

Training a GPR model over a dataset of ***D*** = {***X***, ***y***} made of *N* observations is a challenging task that requires finding the optimal set of model hyperparameters (***θ̂****θ*_*k*_) and the regularisation noise (*σ*_*n*_^2^). Here we rely on the iterative hold-out cross-validation (IHOCV)^20^ approach to determine these hyperparameters. The IHOCV protocol proceeds by minimizing the predictive root-mean-square error of intermediary models over a sufficiently large internal validation set.6
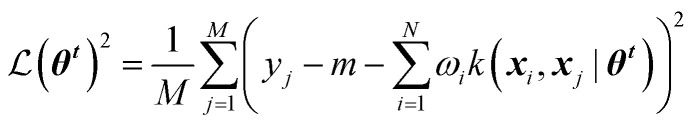
where 
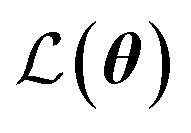
 is the loss function, *m* is the prior mean function of the GP, ***θ***^***t***^ is a candidate solution (set of temporary hyperparameters), and *M* and *N* are the number of validation and training geometries, respectively. Without any loss of generality, *m* is chosen here to be constant and equal to the arithmetic mean of the target property values 
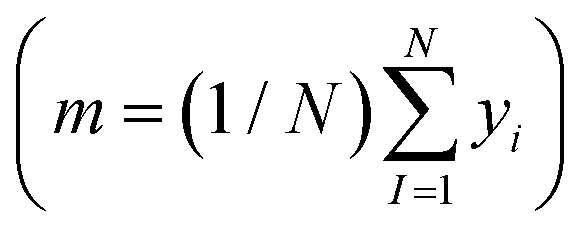
 . Such a mean function guarantees the physicality of all predictions^30^ even in the extrapolation regime.

The coefficients *ω*_*i*_ in eqn (6) are regression weights. These parameters are collected in the weights vector *ω* calculated as shown in eqn (7), where *y*_*m*_ is a mean-shifted vector of target properties, *i.e.****y*** − 1*m*, and ***K***_*XX*_ is the covariance matrix defined in eqn (8),

7 ***ω*** = ***K***_*XX*_^−1^***y***_***m***_

8 *K*_*ij*_ = *k*(***x***_*i*_, ***x***_*j*_|***θ***_*k*_) + *δ*_*ij*_*σ*_*n*_^2^

The term *σ*_*n*_^2^ is a regularisation term often associated with the noise level in the training data, while *δ*_*ij*_ is the Kronecker delta. The symbol *k* represents a covariance function or kernel. This function is meant to measure the similarity between any pair of geometries. The quantity *k*(***x***_*i*_, ***x***_*j*_|***θ***^***t***^) in eqn (6) records the similarity between the *j*th validation geometry and *i*th training geometry.

Every data point (geometry) in the input space is encoded in a vector of fixed length ***x*** using the so-called ALF (atomic local frame) representation,^31^ while similarities between geometries in the ALF space are described using the composite kernel defined in eqn (9),9
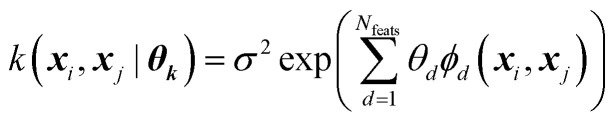
where *N*_feats_ is the number of input features, *d* is the *d*th dimension of the input space and ***θ***_*k*_ is a set of parameters that control the smoothness of the kernel. The stationary function *ϕ*_*d*_ is given dimension-wise by:10
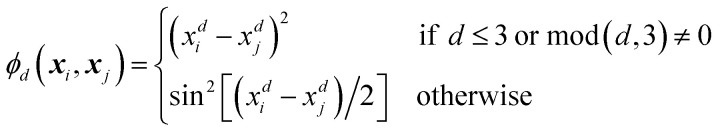
where the function mod(*d*,3) returns the remainder of the division of *d* by 3.

Assuming a GPR model has already been trained, predictions can be made for any new geometry x* using eqn (11),11
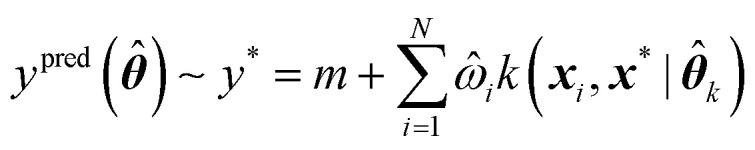
where ***θ̂***_*k*_ is a set of optimal kernel parameters and *ω̂*_*i*_ is the optimal regression weight associated with the *i*th training geometry.

### 2.3. Context and related work

The concept of transfer learning (TL) has a long history in the deep learning community.^32,33^ Despite the peculiarities of each proposed TL protocol, they are all characterized by the sharing of knowledge between domains and corresponding tasks. A typical scenario is one in which prior knowledge about one or several source tasks is leveraged to improve the learning process of a target learner. This setting deviates from the direct or traditional learning paradigm where models are built directly on the target domain.

Transfer learning has shown tremendous success in numerous fields, including computational chemistry,^34^ computer vision,^35^ and natural language processing,^36^ to cite only a few. The TL idea is often resorted to when it comes to (i) improving the predictive capability of (target) regression models and/or (ii) accelerating their training process. The concept has allowed computational chemists to address the scarcity of high-quality data, such as CCSD(T) energies and atomic forces. In general, a big source model, most often a neural network, is trained on a myriad of lower-quality data, and the knowledge (weights and biases) accumulated into the previous source model is leveraged to reduce the risk of overfitting the small high-quality training data.^37^ The training (readjustment) of the target model thus benefits from the existing knowledge, such as physical trends and positions of stationary points, already captured by the large source model. Most importantly, only part of the ML architecture has to be updated, which can save a lot of CPU time.

Rather than focusing on explicitly improving the predictive capability of a given model, our TL protocol (presented in the next paragraph) aims at speeding up the training process of a large target anisotropic GPR model while maintaining the quality of an equivalent but bad scaling DL model. By equivalent we mean a model trained on the same (number of) geometries. Our protocol accelerates the construction of target GPR models by transferring guess hyperparameters from one (or possibly several) smaller source models.

Related to our work are two previous papers that need to be mentioned here. In the first paper,^38^ the authors state that anisotropic kernel parameters optimized on a small dataset could be used unchanged to build a final model over a bigger training set. This argument translates to what we have re-branded “frozen-seed” TL (see Section 2.4.1). Although it makes sense, this assumption ignores the discrepancies between the source and target domains. In some contexts, this can lead to a significant loss of accuracy as compared to an equivalent DL model. To prevent this, our approach allows the hyperparameters learned on the source domain to relax on the target training set, for a few iterations.

The second paper dates back to 2017.^39^ In this work, different authors apply the golden-section search algorithm^40^ to locate an isotropic approximate solution, which they then utilize as the starting point for a gradient-based optimization in the actual anisotropic space. Their gradient-enhanced kriging was shown to achieve better results as compared to random initialization. Like our protocol, their workflow involves two phases. However, unlike us, they choose to unjustifiably ignore the heterogeneity inherent to the input space during the first step, yet without prior feature scaling. Furthermore, their protocol does not incorporate any regularisation noise in the first phase, a choice that exposes it to high risks of numerical instability.^40^ In contrast, our protocol works entirely in the actual HS, with tuneable regularisation noise.

### 2.4. Transfer learning of hyperparameters

#### 2.4.1. The protocol

**Fig. 1:**
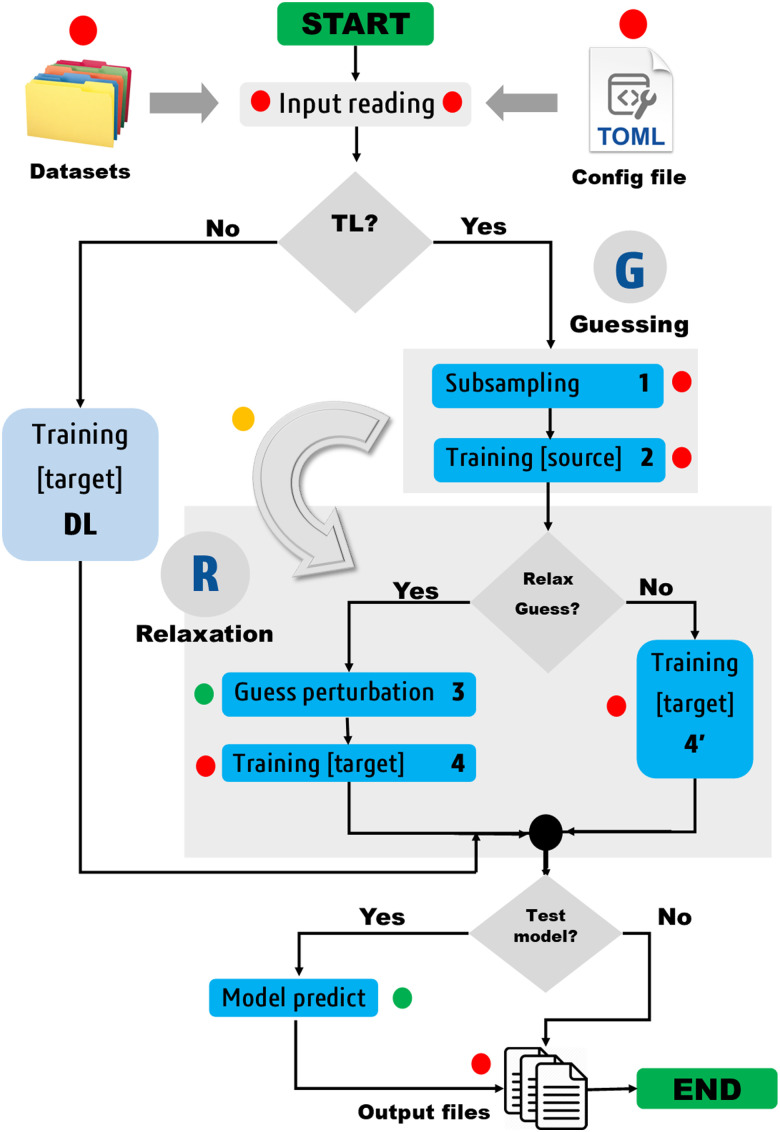
Simplified workflow of the proposed TL/DL protocol. The TL steps are numbered from 1 to 4, while the TL phases are indicated by *G* and *R.* The green- and red-filled circles respectively denote optional and mandatory steps. The perturbation of the guess solution is optional; it is skipped in the case of FS-TL. The yellow-filled circle marks the transfer point. On input, the protocol requires various input files, including dataset files and a configuration.toml file. The latter must be edited by the user to specify control parameters for their job. FEREBUS users can switch the TL pipeline on and off by setting the transfer_learning directive to 1 or 0. Most control parameters have default values defined in the *config.f90* module.

Our protocol (see workflow in Fig. 1) involves four main steps or operations regrouped into two consecutive phases: the seeding or guessing phase 
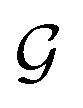
 and the relaxation phase 
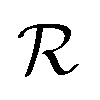
.
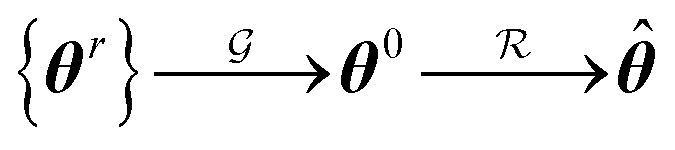
where {***θ***^r^} is a random set of candidate solutions, ***θ***^0^ and ***θ̂*** are the guess and the best achievable (optimal) solutions, respectively.

Two types of models are involved in the design of the protocol: a source model and a target model. The source model is trained on a subset *S* of the target dataset *T*. We propose the name knowledge compression coefficient *η* for the ratio between the sizes of the datasets *S* and *T*. A second control parameter called relaxation weight ζ is also defined as the ratio between the number of relaxation iterations *χ* and the total number of iterations *τ*.

12 *η* = |*S*|/|*T*|

13 *ζ* = *χ*/*τ*

The notation TL^*ζ*^_*η*_ is introduced to denote a transfer learning task defined by the two control parameters *η* and *ζ*. Depending on the value of *ζ* , one can distinguish two special cases: (i) *ζ* = 1 and (ii) *ζ* = 0 . The first case leads to fully seeded TL, while the second case gives rise to a class of unrelaxed models that we have coined frozen-seed transfer learning (FS-TL) models. The notation FS-TL_*η*_ will sometimes be used to denote FS-TL models.

##### Guessing phase 
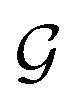


###### Step 1: subsampling

A source training set *S* is generated by random subsampling of the target training set *T*. Besides random selection, FEREBUS is equipped with two enhanced sampling techniques, namely passive and stratified sampling. Both techniques have been widely discussed elsewhere.^41^

###### Step 2: training of source model

Once the dataset *S* has been generated, a source model is trained over it using our grey wolf optimizer described in Section 2.5. For a single-source TL task, this process starts with an initial pool of random solutions {***θ***^r^} and returns a unique guess solution (***θ***^0^). In the case of multisource TL, *N*_s_. solutions are collected and the transfer of these solutions to the target model is briefly discussed in Section 1 of the ESI.†

##### Relaxation phase 
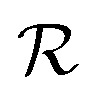


###### Step 3: guess perturbation

For single-source TL such as those examined in this work, only one guess solution is available at the end of phase 
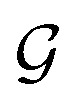
. Since our metaheuristic optimizer requires *W* agents (candidate solutions) to scrutinize the HS, with *W* > 3, phase 
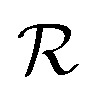
 starts by re-populating the matrix ***P*** of candidate solutions. For this purpose, we first transfer ***θ***^0^ to ***P***, then generate the remaining *W* − 1 candidate solutions (***θ***^0^_pert_) *via* restricted small random walks around ***θ***^0^. The *d*^th^ component of each perturbed solution is computed using eqn (14),

14 ***θ***^0^_pert,*d*_ = ***θ***^0^_*d*_ (1 + *r*_max_*ε*)

where is *ε* a random number uniformly sampled within the range [−1,1] and *r*_max_ is a parameter that controls the extent of perturbations around each component of the vector ***θ***^0^.

By default, *r*_max_ is set to 0.25 (the same value is used in this work). However, the optimal value of this parameter is problem-dependent and should be adjusted such that the ***θ***^0^_pert_ solutions are of similar to better quality than ***θ***^0^. Large *r*_max_ values may lead to solutions of poor quality (situated far from ***θ***^0^), while very small values can restrict the accessible region of the HS. We recommend that FEREBUS users perform a grid search between 0.10 and 0.50 (step of 0.05) to find the most appropriate value for their system and choose the value delivering the best models.

###### Step 4: training of target model

Once ***P*** is available, the algorithm can proceed to the final step, *i.e.*, training the target model.

#### 2.4.2. Timings and speedup

The training time (*Ω*_TL_) of a given TL^*ζ*^_*η*_ model can be decomposed as the sum of the guessing and relaxation timings (
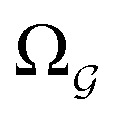
 and 
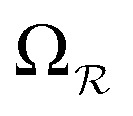
).15
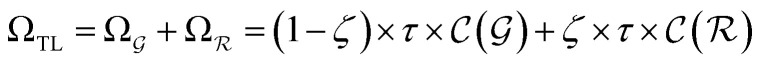
where 
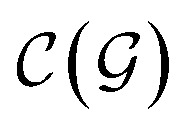
 and 
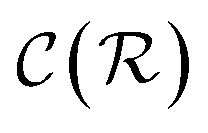
 are respectively the complexity/cost of a single iteration within the guessing and relaxation 
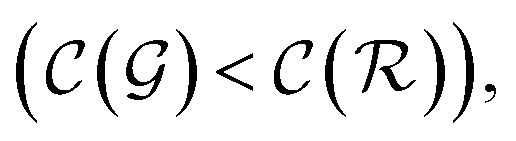
 and *τ* the total number of iterations. By default, phase 
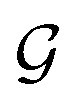
 lasts (1 −ζ) × *τ* iterations, while phase 
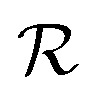
 is propagated for ζ × *τ* iterations. This behavior can be changed by setting the full_seeding directive to 1, in which case the guessing phase runs over *τ* iterations.

In general, 
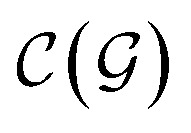
 and 
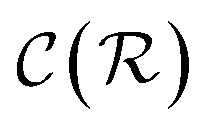
 are dominated by the inversion of the associated covariance matrices, which scale as 
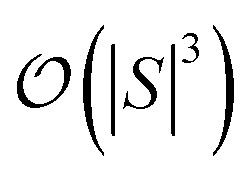
 and 
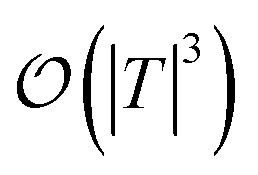
, respectively. Keeping in mind that 
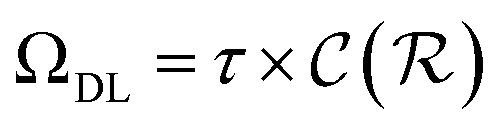
 and neglecting input/output delays one obtains:16
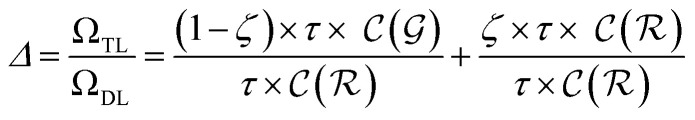


The previous equation can be written more concisely as:17
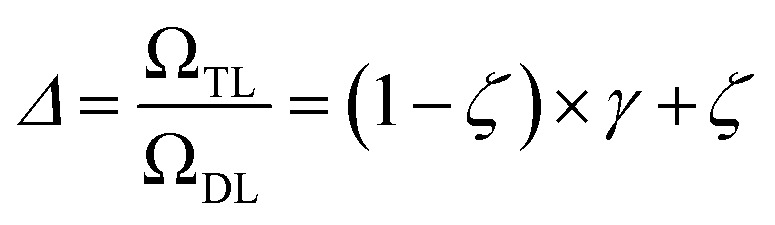
where 
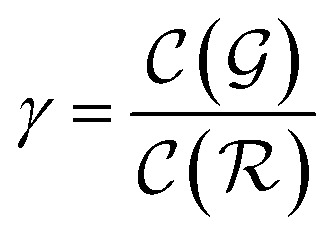
 is the relative complexity of a single training iteration in the guess and relaxation phases of a TL^*ζ*^_*η*_ task.

We distinguish two asymptotic cases depending on the choice of *η*: (i) |*S*| = |*T*| and (ii) |*S*| ≪ |*T*|. In the first case, *γ* = 1 such that Δ = 1. This implies that the TL protocol reduces to DL when *S* is set to be the same as *T* (*η* = 1). In the second case, *γ* ∼ 0 and *Δ* → *ζ*. In this scenario, the expected speed-up (*Δ*^−1^) is upper-bounded by *ζ*^−1^, the inverse of the relaxation weight. In between these two extreme cases, the TL protocol guarantees acceleration if and only if *Δ* < 1, *i.e.* from eqn (17),

18 (1 − *ζ*) × *γ* + *ζ* < 1 ⇒ *γ* < 1

The previous condition is always true in the case of single-source TL^*ζ*^_*η*_ tasks. This is because *S*⊂*T* implies that 
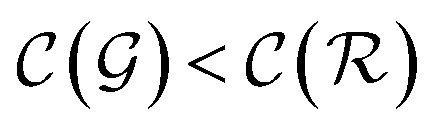
 and *γ* < 1. However, in the case of multi-source TL^*ζ*^_*η*_ tasks, the speed-up condition becomes:19
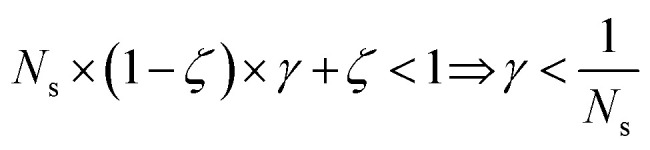
where *N*_s_ is the number of source models built in serial.

A final scenario to consider here is when the full_seeding flag is activated. This flag sets *ζ* to 0 in the guessing phase only, leading to a total of (1 + *ζ*) × *τ* iterations including *τ* steps in phase 
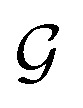
 and *ζ* × *τ* steps in phase 
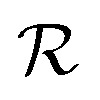
. By setting *ζ* to 0 in the first right-hand term of eqn (16), the speed-up conditions for single-source and multi-source TL models become *γ* < 1 − *ζ* and *γ* < (1 − *ζ*)/*N*_s_, respectively.

### 2.5. GWO-RUHL: an enhanced grey wolf optimizer

The choice of optimizer is a determinant factor of the performance of our TL protocol. FEREBUS relies on our recently reported enhanced grey wolf optimizer (GWO-RUHL)^42^ because of its excellent exploration and exploitation capabilities.

Like vanilla GWO,^43^ the GWO-RUHL algorithm is a metaheuristic optimizer inspired by the predation mechanism and leadership hierarchy of grey wolves. This optimizer utilizes a team of *W* agents to scrutinize the HS. Every agent is encoded as a vector of the same dimension as the HS and constitutes a candidate solution. At the end of each iteration, all candidate solutions are ranked in ascending order of proximity to the optimal solution. The *α*, *β*, and *δ* agents are the best solutions or leaders. Their positions serve to orient the movement of non-leader solutions (called *ω* solutions) toward more promising regions of the search space. The position of an *ω* solution *j* is updated following eqn (20)–(22),

20 *D*_*l*,*d*_ = |*C*_*l*,*d*_·***θ***_*l*,*d*_(*t*) − ***θ***_*j*,*d*_(*t*)|

21
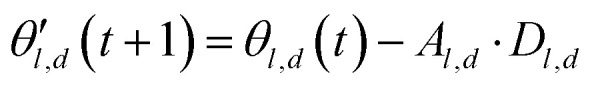
22
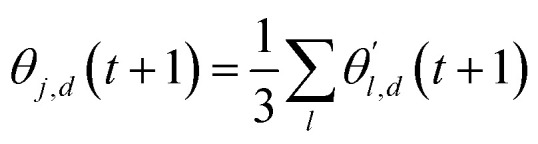
where *t* stands for the current iteration, *l* ∈ {*α*, *β*, *δ*} and *d* is the *d*th dimension of the HS. The stochastic perturbation matrices *A* and *C* are defined in eqn (23) and (24), where *r*_1_ and *r*_1_ are two random numbers in the range [0,1]. The time-dependent parameter *a*(*t*) is decreased linearly from *a*_max_ to 0 to control the balance between exploration and exploitation of the HS during the optimization process.

23 *A*_*l*,*d*_(*t*) = 2*a*(*t*)*r*_1_ − *a*(*t*)

24 *C*_*l*,*d*_(*t*) = 2*a*(*t*)*r*_2_

Improving over vanilla GWO, our GWO-RUHL(*n*,*p*) algorithm (*n* and *p* are explained below) accounts for the natural desire of ω wolves to occupy high-ranked positions in the leadership hierarchy. This is achieved by inserting in the previous search mechanism a new operator *Û*, which acts on the current population ***P*** and promotes a certain number *n* of *ω* solutions toward new positions situated in the vicinity of the centroid of the three leaders (positions believed to host better solutions). This operation, *ÛP* = ***P***′, is repeated every *p* iteration and each promoted (lucky) solution is calculated using eqn (25),25
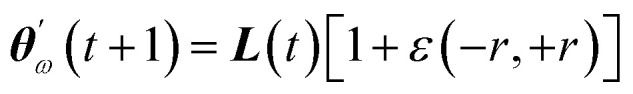
where *ω* is a randomly selected (lucky) non-leader wolf (solution), ***L*** is the centroid of the leaders' positions, and *ε*(−*r*, +*r*) is a random number between −*r* and +*r* (with *r* ∈ [0,1]).

## 3. Computational details

### 3.1. Conformational sampling

The initial datasets were made of 10 000 geometries of each of the four molecules of interest. Geometries of BZ and ETL were retrieved from the original MD17 database,^44^ while those of FAD and FPL were obtained through unbiased semi-empirical molecular dynamics (USEMD) simulations.

**Fig. 2:**
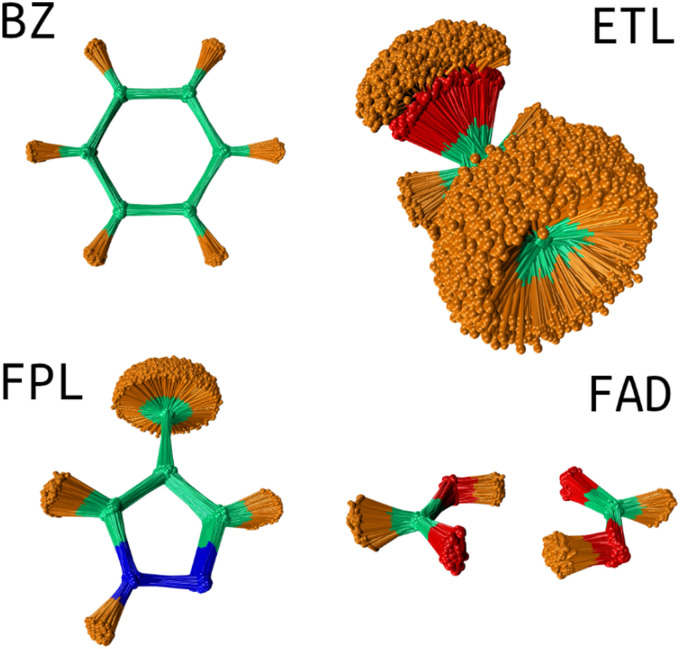
Conformational space sampling: the overlaid geometries were rotated about the first trajectory frame using the Kabsch algorithm.^46^ This image shows the pronounced flexibility of the ETL molecule allowing for full rotation of the CH_3_ and OH groups. The sampled conformation space of FAD involves stretching of the two hydrogen bonds and movements about the plane of the molecule. BZ is very rigid while FPL shows fairly considerable structural fluctuations around the CH_3_, CH and NH groups.

USEMD simulations were performed in the gas phase without periodic boundary conditions using the GFN2-xTB method^45^ as implemented in the atomic simulation environment (ASE) Python package.^4^ Each simulation was performed in the canonical NVT ensemble at 300 K using the Langevin thermostat with a friction coefficient of 0.01 fs^−1^. All simulations were propagated for 1 ns with a timestep of 1 fs. Snapshots were retrieved every 100 fs leading to a generous population of 10 000 geometries. Fig. 2 shows the conformational space sampling of each molecule of interest.

### 3.2. QTAIM/IQA calculations, featurisation, and dataset filtering

Once collected, all the geometries were passed to our in-house ICHOR^31^ package. The latter is a Python package that automates the calculation of both input features and target properties. Wavefunctions were extracted *via* single-point calculations at B3LYP/6-31+G(d,p) (FAD and FPL) and B3LYP/aug-cc-pVTZ (BZ and ETL) levels of theory using GAUSSIAN16.^47^ Each wavefunction was then processed by AIMAll19^48^ to compute the atomic QTAIM/IQA properties using equations described in Section 2.1. Concomitantly, each geometry was encoded as a vector of fixed length using the so-called atomic local frame (ALF) molecular descriptor.^31^ The resulting features were combined with the target properties (IQA and *Q*_00_), leading to an initial database that was later filtered by removing all geometries for which the molecular energy and charge could not be reconstructed within margins of 1 kJ mol^−1^ and 1 me. The filtered database was randomly split into training, validation, and test sets. A summary of the filtering results is provided in Section 2 of the ESI.†

### 3.3. Model construction and optimization settings

All atomic GPR models were trained following the IHOCV protocol using 8 cores on compute nodes equipped with Cascade Lake Xeon Gold 6230 CPUs of 2.10 GHz clock speed each. The smallest DL models (1000 training geometries) were also retrained using 1, 4, 12, 16 and 20 cores to assess the effect of computing resources on the building cost.

Preliminary experiments were conducted to determine the validation set size and the number of candidate solutions that provide a reasonable trade-off before accuracy, cost, and consistency of predictive metrics upon successive runs. The results obtained for the smallest direct learning (DL) models suggested that a validation set of 500 geometries produced the best models. All the models were tested on 1000 geometries.

Optimization runs were carried out for a maximum of *τ* = 200 iterations using a set of 50 active agents identified among an initial random population of 500 candidate solutions thrown on the HS. The following control parameters were chosen for the GWO-RUHL(*n*,*p*) algorithm: *a*_max_ = 2.0 (except in phase 
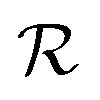
 of all TL^*ζ*^_*η*_ tasks where *a*_max_ was set to 1.0 to promote the exploitation/intensification of the HS43), *r* = 0.20, *n* = 5 and *p* = 5. As per *n* and *p* values, 5 non-leader solutions were promoted every 5 iterations toward promising regions of the HS, whose boundaries were defined as [0.0,3.0] and [10^−14^,10^−4^] for the kernel parameters and regularisation noise, respectively. To avoid unphysical solutions, FEREBUS makes sure all solutions crossing the walls of the previous HS are randomly reinitialized inside the boundaries of the HS during the optimization process.^49^ All FEREBUS control parameters referred to in this paper are described in Table S1 of the ESI.†

Before we compare the performance and training costs of DL and TL models, we took care to first assess the training cost and learning capability of DL models. For this purpose, we monitored the training timings and predictive mean absolute errors (MAEs) of each DL model as we varied several factors, including the number of training points (from 1000 to 8000 geometries) and the validation set size (between 25 and 500 geometries). We made sure these datasets did not overlap with each other or with the test set.

After careful examination of DL models, we moved on to building and assessing the performance of TL models. Single-source TL models were built by targeting the largest DL models. For each atom and target property, we trained 9 = 3 × 3 different single-source TL models for all combinations of *η* (0.00, 0.10, 0.25) and *ζ* (0.00, 0.05, 0.10) parameters. Unless otherwise stated, (i) guess solutions were perturbed using a maximum deviation parameter *r*_max_ of 0.25, and (ii) no prior scaling of the features and target properties was applied.

By design, FEREBUS builds atomic GPR models. However, molecular predictions can be easily obtained through reconstruction using eqn (26),26
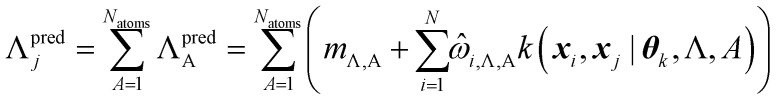
where *j* denotes a given test molecule, *Λ* is the target property (either *E*_IQA_ or *Q*_00_), *N* is the number of training geometries and *N*_atoms_ is the number of atoms in molecule *j*. All the other terms have the same meaning as in Section 2.2.

### 3.4. Normal mode calculations

Normal mode calculations were carried out for each molecule of interest. The GAUSSIAN optimized geometry (at the reference level of theory specified in Section 3.2) was slightly perturbed by moving each atom 0.01 Å back and forth in the *XYZ* directions. Then, atomic forces were computed using analytical formulae implemented in DL_FFLUX. Using the previous atomic forces and the perturbed geometries, normal modes, and vibrational frequencies were computed using the finite-difference method implemented in Phonopy.^50^ These calculations involved the diagonalization of a mass-weighted Hessian matrix whose eigenvectors and eigenvalues identify with the normal modes and vibrational frequencies, respectively.

### 3.5. Geometry optimisation

The program DL_FFLUX is built on DL_POLY 4^51,52^ and shares many of its routines. One of these is the “Zero Kelvin” optimizer thanks to which DL_FFLUX can perform geometry optimization calculations by maintaining the velocity of atoms below 10 K during a short simulation. In this way, the movement of atoms follows the direction of calculated forces and torques. DL_FFLUX geometry optimization calculations were propagated for 2 ps with a timestep of 1 fs in the NVT ensemble using the Nosé–Hoover thermostat and a relaxation time of 0.2 ps. The starting geometries ***q***^0^ were carefully generated by perturbing the GAUSSIAN minimum (***q***_min_) along its normal modes. For each normal mode ***q***, ***q***^0^ was defined as shown in eqn (27),

27 ***q***^0^ = ***q***_min_ + DF × ***q***

where the displacement factor (DF) is chosen to vary between 0.1 and 0.5.

## 4. Results and discussion

### 4.1. Performance of DL models

#### 4.1.1. Learning curves

In this section, we discuss the performance of direct learning (DL) models. The largest of these models were trained on 8000 geometries, which will serve as references when discussing the performance of TL models in Section 4.2. We emphasize that all the models reported here were trained using an anisotropic kernel in the context of the IHOCV approach. This choice is justified in Section 4 of the ESI† (Table S2).

**Fig. 3:**
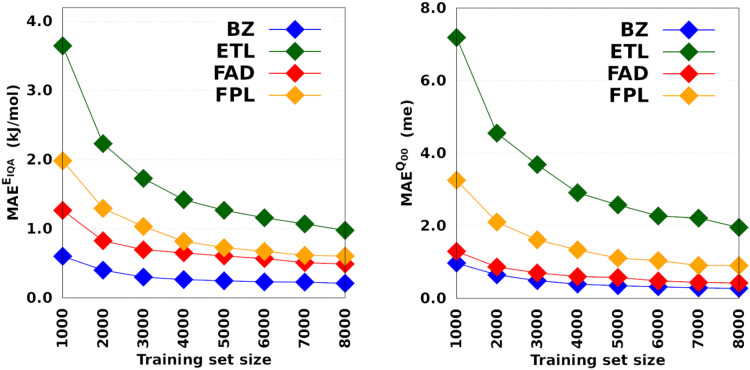
Learning curves of anisotropic GPR models trained on 1000 to 8000 geometries. All the models were tested on the same set of 1000 geometries. The hyperparameters were optimized on an internal validation set of 500 geometries.

Fig. 3 depicts the molecular learning curves of DL models. The corresponding element-wise learning curves and molecular learning *S*-curves are collected in Section 5 of the ESI.† Notice that the curves in Fig. 3 are well-behaved^53^ in the sense that the models progressively and consistently improve their generalization aptitude as we increase the training set size. Such learning curves are characteristics of well-posed learning problems and are sometimes called monotonic^54^ curves. Theoretical studies on the link between GP assumptions and learning behavior suggest that ill-behaved learning curves (with bumps) could be indicative of a misspecified GP.^55^

The performance of *E*_IQA_ models improved by a factor of 2.9, 3.7, 2.6, and 3.3 as we augmented the training set from 1000 to 8000 geometries in the case of BZ, ETL, FAD, and FPL, respectively. Similar refinement factors of 3.5, 3.7, 3.1, and 3.6 were respectively observed for *Q*_00_ models. The fact that the predictive MAEs of the largest DL models lie within the limit of chemical accuracy (∼4 kJ mol^−1^) advocates for the adequateness of these models to be deployed in ML-aided simulations. Furthermore, except for ETL, all the largest DL models managed to reproduce molecular charges with predictive MAEs below 1 me.

The learning curves in Fig. 3 demonstrate the peculiarity of each system in terms of performance, learning slopes/rate and saturation point location. These overall and unique features reflect the different molecular flexibilities and levels of complexity of the conformational spaces under investigation. For instance, the fact that BZ is a very rigid molecule justifies the outstanding ability of the DL models to reproduce its atomic properties. In contrast, despite its smaller size, ETL is relatively more flexible than BZ and this attribute is mirrored by the higher predictive MAE values observed for DL models of ETL compared to those of BZ. From another perspective, the same reasoning explains why the local properties of atoms that move more tend to be more difficult to machine-learn.

Finally, it is worth emphasising that static performance metrics (such as the MAE values reported in this section) are not always conclusive. Since the training and test sets come from the same dataset, they inherit its caveats. For this reason, the computed MAEs cannot tell how robust a model is, that is, how it will behave in a real deployment scenario such as in a FFLUX simulation. One must then be careful when inferring conclusions from such metrics. In particular, this is true when it comes to guessing the relative stability of MLP-driven simulations, which is mostly a matter of the extent of the conformational space coverage.

#### 4.1.2. Training time

While data augmentation improves the predictive capability of a model, it also comes at the price of an increased computational training cost. In this section, we investigate how the training cost of DL models by FEREBUS only is influenced by the training set size, the amount of resources available, and the validation set size. Herein, we evaluate the training cost in terms of wall time and CPU times. Of course, the wall time reflects what the user perceives, while the CPU time relates to the amount of computer power used by a program.^56^ In its standard output file, FEREBUS decomposes the CPU time into the user time and the time devoted to system calls.

**Fig. 4:**
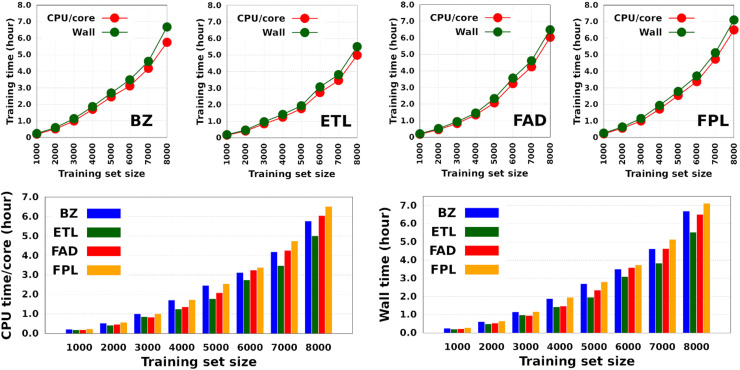
Training cost of anisotropic GPR models of atomic energies and charges. CPU/core and wall times expressed in hours. Individual timings for each molecule are shown in the top panel, while the bottom histograms combine individual data to facilitate comparison.

Fig. 4 shows the CPU/core and wall times of atomic GPR-DL models trained on 1000 to 8000 geometries. The raw data are collected in Table S3 in Section 6 of the ESI.† The timings reported in this study correspond to the shortest CPU/core and wall times recorded for a set of *E*_IQA_ and *Q*_00_ models trained on the same geometries. Comparing the shortest timings is meant to alleviate the undesired and biasing effect of architecture-related noise.

According to Fig. 4, the training cost increases monotonically with the size of the training set. For a fixed model size (number of training geometries), it is the dimension of the HS (*N*_feats_ + 2) that controls the training cost. This explains why training the largest DL model of FPL (*N*_feats_ = 30) took 90 minutes longer than ETL (*N*_feats_ = 21). In general, CPU/core and wall times increase in the order ETL < FAD < BZ < FPL. A slightly different pattern is observed for models trained on 6000 to 8000 geometries, where the training costs of BZ and FAD models are swapped. We attribute this effect to the datasets themselves, more specifically to how the relative positions of geometries in the input space affect the condition number of the covariance matrix. Indeed, adverse effects of ill-conditioning can be amplified by the dimension of the covariance matrix, which may have been the case between FAD and BZ models as we kept extending their knowledge content.

**Fig. 5:**
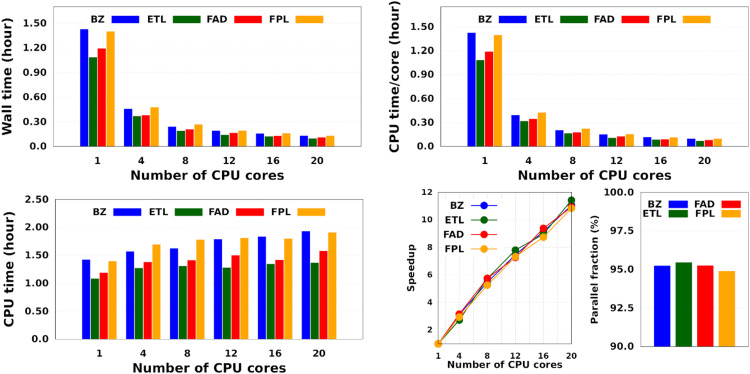
Effect of the number of CPU cores on the training cost of anisotropic GPR models. These results are those of the smallest GPR models. Qualitatively equivalent trends are expected for larger models.

Intuitively, one should expect the training time of anisotropic GPR models to also depend on the number of computing resources available at runtime. Fig. 5 shows the training CPU/core and wall times of DL models trained on 1000 geometries using an increasing number of CPU cores, namely 1, 4, 8, 12, 16, and 20 cores. We reiterate that the results reported elsewhere in this work were obtained using 8 cores. It turns out that, unlike the total CPU time, which is less sensitive to the number of cores requested per training job (random variations due to system calls), the wall times (and the CPU time per core) decrease almost linearly with the number of CPU cores engaged in the training process. Notice also that for the smallest training set, the CPU/core and wall times differ by up to 17%, which suggests that in these cases, the program spent a non-negligible portion of the wall time dealing with non-CPU-related delays, such as waiting for resources to be available. Furthermore, the fact that the speedup increases monotonically (yet not linearly) with the number of CPU cores is a strong argument in favor of the reasonably good parallelization of our GPR engine (with ∼95% parallelization according to Amdahl's law^57^). We reiterate that FEREBUS relies on OpenMP to distribute the workload over several threads in the hottest parts of the code.^18,19,42^ These hot spots correspond to routines that compute the loss functions for each candidate solution.

**Fig. 6:**
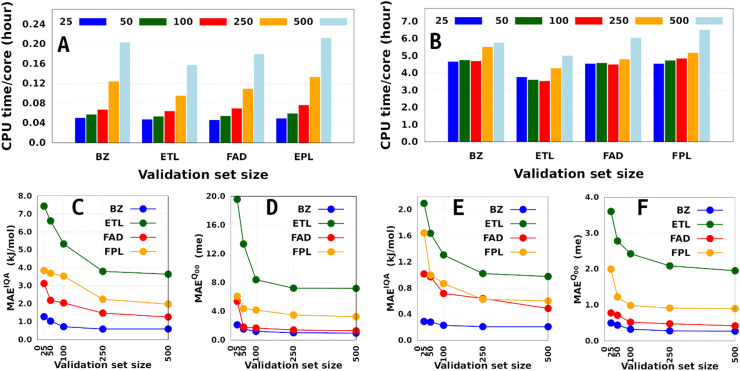
Effect of the validation set size on the training cost (A) and (B) and performance (C) and (D) of DL models of BZ, ETL, FAD, and FPL training using 1000 (A), (C) and (D) and 8000 (B), (E) and (F) geometries.

Besides the training set size and the computing resources available, the size of the fixed validation set is another important factor that determines both the generalization aptitude and training cost of anisotropic GPR models within the IHOCV framework. Fig. 6 illustrates the training costs (CPU times per core) and predictive MAEs of various DL models trained on 1000 and on 8000 geometries using an increasing number of validation geometries (25 to 500). In general, it is right to think that the training time will always increase with the number of validation geometries. However, we find that the extent of this effect is both system-dependent and inversely proportional to the size of the training set. Notice that the smallest DL model is more affected than the largest DL model, with overhead factors of up to 4.3 and 1.4, respectively. The fact that the validation-induced overhead decreases with the training-validation split encourages, when affordable, the selection of larger validation sets dealing with large training sets. Additionally, because larger validation sets are more representative of the data being machine-learned, they tend to produce models that do not overfit them and generalize better on the test set.^58^ We note in passing that model overfitting is a general problem in machine learning diagnosed when a model overestimates its generalization capability, thus performing poorly on the test set. Although more pronounced in artificial neural networks due to a large number of parameters,^38,59,60^ it also affects GPs.^61^

Finally, although offering non-negligible speed-up (especially on small training sets), validation sets containing less than 100 geometries seem inappropriate as far as the generalization aptitude of the final model is concerned. However, we must emphasize that the optimal size of a fixed validation set is problem-dependent and is likely to change with the test set size, the complexity of the conformational space, and the chosen data sampling technique. Talking of the latter, it is to be anticipated that enhanced sampling techniques such as adaptive sampling^62^ could help reduce the size of an optimal validation set by selecting the most informative geometries from the initial sample pool. Unfortunately, applying such techniques often comes at the price of higher computational cost, which needs to be examined carefully to appreciate the significance of their improvement as compared to random selection.

### 4.2. Performance of TL models

#### 4.2.1. Transfer learning of hyperparameters does work

In this section, we demonstrate the effectiveness of the transfer learning of hyperparameters. For this purpose, we compare three single-source FS-TL models TL^0.00^_0.01_, TL^0.00^_0.10_ and TL^0.00^_0.25_ with two types of baseline models. In the notation TL^*ζ*^_*η*_, the control parameter *η* represents the knowledg*e* compression coefficient while *ζ* is the relaxation weight. The first baselines were built using 1% (DL^1%^), 10% (DL^10%^) and 25% (DL^25%^) of the largest training set, corresponding to 80, 800, and 2000 geometries, respectively. These local GPR models were trained on the same number of geometries as the source domains of the FS-TL models. The second type of baseline DL models were built using the best solution among 500 randomly generated candidate solutions. These models were initialized on 8000 training geometries but the hyperparameters were not optimized. We will use the acronym RBL to refer to these random baseline models.

**Fig. 7:**
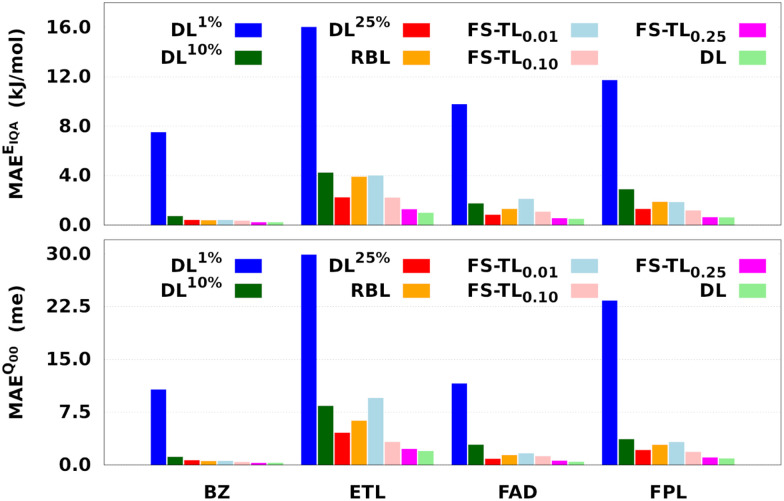
Predictive MAEs of *E*_IQA_ and *Q*_00_ baseline and FS-TL models of BZ, ETL, FAD, and FPL. We only examine the reconstruction of molecular quantities to obtain an overall appreciation of the quality of atomic GPR models. The models TL^0.00^_0.01_, TL^0.00^_0.10_, and TL^0.00^_0.25_ are respectively labeled as FS-TL_0.01_, FS-TL_0.10_, and FS-TL_0.25_.

Fig. 7 and Table 1 respectively report the predictive MAEs and training costs of the baseline and FS-TL models. The raw predictive MAEs and acceleration factors are collected in Tables S3 and S4 (ESI†). It turns out that FS-TL models always outperform their local GP analogs when it comes to reconstructing both the molecular IQA energies and charges. Fig. 7 and Table S4 (ESI†) suggest that the predictive MAEs of TL^0.00^_0.01_ models can be an order of magnitude lower than those of DL1% ones, with the highest deviation factors of ∼19 recorded for BZ. However, as one extends the size of the local GPs, their performance converges toward that of equivalent FS-TL models, whose enhanced performance is due to the explicit awareness of the target domain.

**Table 1:** Training timings of the baseline and FS-TL models of BZ, ETL, FAD, and FPL. All timings are expressed in hours and correspond to the shortest CPU/core and wall time recorded for a set of models trained on the same dataset. For comparison purposes, we also indicate the training time of the largest DL model

Model	CPU time per core				Wall time			
	BZ	ETL	FAD	FPL	BZ	ETL	FAD	FPL
DL	5.751	4.990	6.033	6.498	6.675	5.508	6.488	7.101
DL^1%^	0.011	0.007	0.009	0.009	0.013	0.009	0.010	0.012
DL^10%^	0.155	0.117	0.144	0.168	0.187	0.141	0.170	0.202
DL^25%^	0.516	0.409	0.455	0.559	0.598	0.467	0.521	0.635
TL^0.00^_0.01_	0.041	0.032	0.039	0.042	0.061	0.047	0.056	0.065
TL^0.00^_0.10_	0.144	0.130	0.129	0.162	0.171	0.158	0.155	0.200
TL^0.00^_0.25_	0.433	0.369	0.428	0.468	0.489	0.433	0.486	0.533
RBL	0.295	0.288	0.332	0.336	0.328	0.318	0.365	0.369

As expected, the predictive capability of FS-TL models improves as the source domain becomes more and more similar to the target domain. For instance, FS-TL_0.01_, FS-TL_0.10_, and FS-TL_0.25_*E*_IQA_ models of BZ (FPL) recovered 53.7% (32.8%), 62.1% (51.9%) and 97.7% (98.2%) of the predictive accuracy of the largest DL model, while respectively achieving speedup factors of 109.4 (109.2), 39.0 (35.5), and 13.7 (13.3). However, we note that seeding FS-TL hyperparameters on a small and non-representative source dataset leads to suboptimal performance. In such cases, the guess hyperparameters are of poor quality and must be refined *via* relaxation on the target domain. More specifically, FS-TL models seeded on less than 10% of the target training set tend to perform significantly worse than the largest DL models, even though doing better than the local GP baselines. In terms of building cost, FS-TL_0.10_ and DL^10%^ models exhibit comparable CPU and wall times, which corroborates the fact that the training of FS-TL models does not involve any expensive relaxation of the guess solution.

On the other hand, we find that the performance of RBL models is always lower than that of TL^0.00^_0.25_ models, while their building cost lies in between those of TL^0.00^_0.10_ and TL^0.00^_0.25_ models. Most importantly, despite consuming two to three times fewer CPU hours than the RBL models, TL^0.00^_0.10_ models perform much better. Additionally, unlike FS-TL models, the quality of RBL models might fluctuate considerably due to the unpredictable nature of random candidate solutions. It also stands to reason to think that the overall quality of such random solutions will generally deteriorate with the size of the molecule (∼dimension of the hyperparameter space) and the complexity of the conformational space. This intuitive, yet justifiable hypothesis disqualifies RBL models as a viable option for the fast construction of anisotropic GPR models.

The above findings suggest that a unique FS-TL model always performs better than any single local GP built on the same subspace of the target dataset as the source model of the FS-TL model. Similarly, FS-TL models, which are aware of the target domain, are expected to perform better than any approximative sparse GP model whose inducing points are the same as the source dataset (geometries) of the FS-TL model. However, we anticipate cohorts of local GPs^16^ trained on smartly clustered subsets of a target dataset to demonstrate enhanced performance, comparable to relaxed TL and full DL models.

#### 4.2.2. DL-*versus*-TL models: performance comparison

We have shown how the transfer of hyperparameters is a viable option for the efficient construction of GPR models. However, we also noticed that when the source model is very small, this practice can lead to sub-optimal models, unless the guess solution is subjected to relaxation. In this section, we examine the effect of source model size and relaxation weight on the training cost and performance of TL models.

**Fig. 8:**
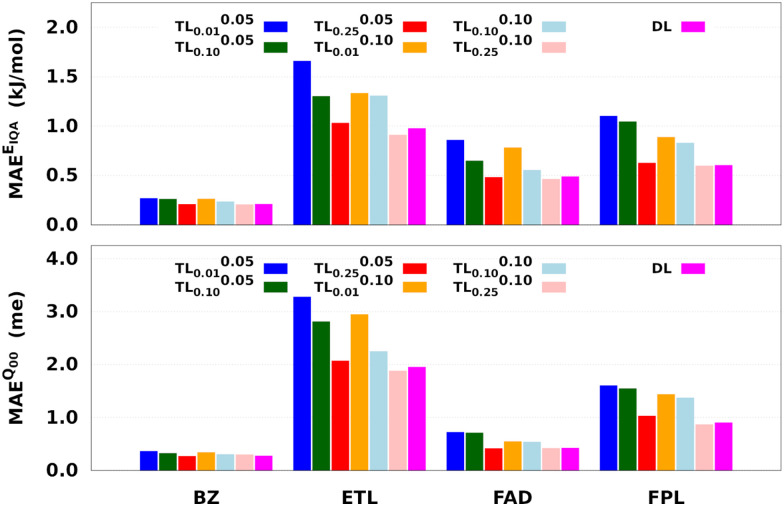
Predictive MAEs of *E*_IQA_ and *Q*_00_ relaxed TL models of BZ, ETL, FAD, and FPL. Also indicated is the performance of the DL reference. We focus on the reconstruction of molecular quantities to obtain an overall appreciation of the quality of atomic GPR models.

Fig. 8 reports the performance of the largest DL and a series of shortly relaxed TL models trained on 8000 geometries using a validation set of 500 geometries. The relaxation weight was kept small (<0.10) to achieve a significant speed-up. Table 2 gives the training timings in terms of CPU time per core and wall time. Tables S5 and S6 (ESI†) respectively collect the raw predictive MAEs and speedup factors of the relaxed TL models.

**Table 2:** Training timings of the largest DL and (relaxed) TL models of BZ, ETL, FAD, and FPL. All timings are expressed in hours and correspond to the shortest CPU/core and wall time recorded for a set of equivalent *E*_IQA_ and *Q*_00_ models trained on the same dataset

Model	CPU time per core				Wall time			
	BZ	ETL	FAD	FPL	BZ	ETL	FAD	FPL
DL	5.751	4.990	6.033	6.498	6.675	5.508	6.488	7.101
TL^0.05^_0.01_	0.340	0.217	0.295	0.358	0.398	0.248	0.330	0.414
TL^0.05^_0.10_	0.420	0.309	0.366	0.445	0.474	0.351	0.399	0.509
TL^0.05^_0.25_	0.728	0.597	0.715	0.773	0.839	0.676	0.785	0.895
TL^0.10^_0.01_	0.667	0.446	0.498	0.668	0.773	0.507	0.554	0.777
TL^0.10^_0.10_	0.679	0.587	0.629	0.674	0.767	0.649	0.687	0.747
TL^0.10^_0.25_	1.077	0.821	0.951	1.121	1.252	0.916	1.070	1.242

Two main observations come out of Fig. 8. First, it can be seen that, for a given relaxation weight, the performance of TL models increases with the size of the source model. Most importantly, TL models trained on 25% of the target dataset and a relaxation weight of 0.10 (20 relaxation iterations) outperform the equivalent DL models. For instance, TL^0.10^_0.25_ models of ETL achieve MAEs of 0.91 kJ mol^−1^ and 1.8 me against 0.98 kJ mol^−1^ and 1.9 me for the largest DL model. Second, we observe that TL^*ζ*^_0.01_ and TL^*ζ*^_0.10_ (*ζ* ≠ 0) models achieve much better predictive accuracy when compared to FS-TL_0.01_ and FS-TL_0.10_ models. This observation confirms the relevance of guess relaxation on small source models.

Table 2 and Table S6 (ESI†) suggest that relaxed TL models can be built five to eight times faster than their DL analogs while preserving the quality of the latter or even outperforming them. It is important to mention that, as long as the size of the source model is large enough, FS-TL models already offer a reasonable cost/accuracy trade-off when it comes to reproducing molecular IQA energies. Indeed, FS-TL_0.25_*E*_IQA_ models are capable of recovering 98% of the largest DL model's performance. However, unlike IQA energies, FS-TL models struggle more when it comes to reconstructing the molecular charge. Even with a source dataset of 2000 geometries, FS-TL_0.25_*Q*_00_ models of ETL, FAD, and FPL respectively recovered 86.7%, 71.6%, and 87.2% of the DL model's performance (less than 90%). Fig. 8 and Table S6 (ESI†) indicate that a short relaxation of the previous FS-TL_0.25_*Q*_00_ guess solutions (for only 10 iterations; *ζ* = 0.05) leads to excellent accuracy recovery rates of 94.3%, 101.9%, and 92.3%.

### 4.3. Prediction of vibrational normal modes

**Fig. 9:**
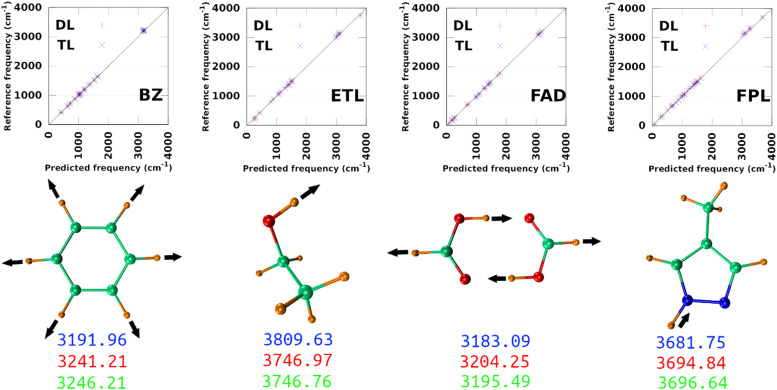
Prediction of vibrational normal modes using DL and TL models of BZ, ETL, FAD, and FPL. The top panel shows scatter plots of reference-*vs.*-predicted frequencies, while the bottom panel shows the highest frequency normal modes. The blue, red, and green numbers are respectively the reference (exact), DL, and TL predicted frequencies. The collective movements of each mode are also depicted.

The largest DL and best-performing single-source TL models (TL^0.10^_0.25_) were employed to compute the harmonic normal modes and frequencies of BZ, ETL, FAD, and FPL in their respective ground states. The predicted frequencies were compared with reference values calculated using GAUSSIAN16^47^ at the appropriate levels of theory (see Section 3.2). Fig. 9 shows scatter plots of the reference frequencies as functions of the predicted ones. The predictive MAEs are collected in Table 3 along with the ID (number) of the worst predicted normal mode and the maximum absolute frequency deviation. Individual vibrational frequencies are collected in Section 9 of the ESI† (Tables S7–S10).

**Table 3:** Performance (MAE and RMSE in parentheses) of DL and TL models in predicting the vibrational frequencies of BZ, ETL, FAD, and FPL. The acronym WPM denotes the worst predicted normal mode ID, while Δ*ν*_max_ stands for the maximum absolute frequency deviation. All frequencies are expressed in cm^−1^. These values can be expressed in energy units using a conversion factor of 0.012 kJ mol^−1^ for each cm^−1^. The quantity *R*^2^ is the Pearson's correlation coefficient between the reference and predicted frequencies

Model	MAE(RMSE)_DL_	MAE(RMSE)_TL_	WPM_DL_	WPM_TL_	Δ*v*_max,DL_	Δ*v*_max,TL_	*R*_DL_^2^	*R*_TL_^2^
BZ	15.21(20.01)	16.18(20.81)	30	30	49.26	54.25	1.00	1.00
ETL	23.25(30.14)	19.02(21.52)	1	1	76.67	63.21	0.99	0.99
FAD	15.05(20.13)	11.92(16.17)	23	23	54.91	42.65	1.00	1.00
FPL	23.99(31.15)	20.57(28.27)	25	29	92.49	75.24	0.99	0.99

As per Table 3, both the DL and TL models achieve decent frequency predictions as compared to the reference levels of theory. In all cases, TL models achieve similar to slightly better average predictions as compared to their DL analogs. The fact that the MAEs of FFLUX-predicted frequencies lie within 100 cm^−1^ (1.2 kJ mol^−1^) of the reference data advocates for the outstanding quality of both series of models. This conclusion corroborates the excellent Pearson's *R*^2^ correlation coefficients between the reference and FFLUX-predicted frequencies.

In general, DL and TL models struggle more with higher frequency modes occurring in the frequency region >3000 cm^−1^. For instance, the worst predicted normal modes of BZ are related to the ring-breathing mode that appears around 3191.96 cm^−1^ at the B3LYP/aug-cc-pVTZ level. The only exception to this pattern is ETL for which the worst predicted mode is the lowest frequency normal mode (263.3 cm^−1^ at the B3LYP/aug-cc-pVTZ level of theory). This unexpected observation can be attributed to a poor sampling along the directions of this normal mode. Interestingly, this observation offers an opportunity to improve the quality of the original DL and TL models *via* data augmentation with carefully sampled geometries along specific eigenvectors of the mass-weighted Hessian matrix.

### 4.4. Geometry optimisation

**Fig. 10:**
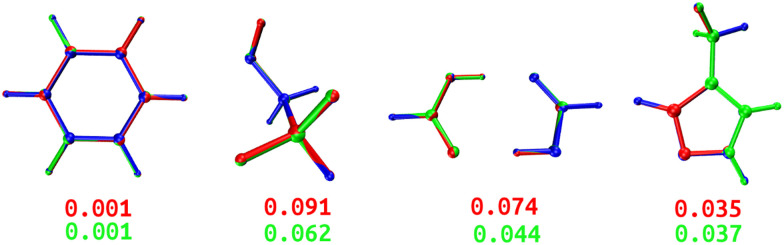
Overlayed optimized geometries of BZ, ETL, FAD, and FPL. The blue-colored structure is the GAUSSIAN reference, while the red and green ones are FFLUX-optimized structures using the DL and TL models, respectively. Both structures were rotated with respect to the reference using the Kabsch algorithm.^46^ The RMSD (Å) between each FFLUX predicted minimum and the reference structure are also shown.

Both DL and TL models were employed in “Zero Kelvin” FFLUX simulations for geometrical optimization. Table 4 collects the average energy difference and root mean square deviation between the GAUSSIAN and FFLUX-optimised geometries. Fig. 10 shows overlayed geometries of the optimized molecules. We also compare in Table S12 (ESI†) the electronic energy range within the starting pool of geometries and the training geometries.

**Table 4:** GPR-aided FFLUX geometry optimization using DL and TL models. Absolute electronic energy differences (Δ*E*) and root mean square deviations (RMSD) between FFLUX and GAUSSIAN-optimised geometries are respectively expressed in kJ mol^−1^ and Å. All optimization runs converged towards structures within 1 kJ mol^−1^ and 0.1 Å from the reference structure

Model	Δ*E*_DL_	Δ*E*_TL_	RMSD_DL_	RMSD_TL_
BZ	0.328	0.275	0.001	0.001
ETL	0.145	0.042	0.091	0.062
FAD	0.070	0.049	0.074	0.044
FPL	0.529	0.371	0.035	0.037

As expected, TL models outperform or at least compete with their DL analogs in terms of energetics and minimum structure prediction. Most importantly, we find that, despite being sometimes launched from geometries located outside the training space, all FFLUX optimization runs using both the DL and TL models outstandingly converged toward the nearest vicinity of the reference minimum structure (Δ*E* < 1 kJ mol^−1^ and RMSD < 0.1 Å). Table S12 (ESI†) indicates that, in all cases, the pool of starting geometries covered a large energy range an order of magnitude bigger than the training set. This finding demonstrates, at least partly, the extrapolation capability of our GPR models.

## 5. Conclusion

The exorbitant training cost of GPs is the main bottleneck that prevents or discourages their application on large datasets. Several solutions have been proposed to address this issue, including reduced-rank approximations and the usage of iterative solvers. However, most of these schemes do not always preserve the desired and outstanding predictive capability of exact (full) GP inference.

In this study, we have proposed a transfer learning (TL) protocol that mitigates the training cost of anisotropic GPR models, while not sacrificing accuracy. The protocol works by transferring hyperparameters from one (or several) small source models to the target GPR model. Performance comparisons between direct learning (DL) and TL models prove that the latter can be trained several times faster while preserving the desired predictive capability of the former. More specifically, TL models trained on 25% of the target training set, and relaxed for 5% to 10% of the total number of iterations, can be built 5 to 8 times faster than their DL analogs, while always recovering more than 90% of the predictive capability of the latter or even outperforming them. We have also shown that, in the case of *E*_IQA_ models, FS-TL_0.25_ models can already recover up to 98% of the DL's performance for an order of magnitude speed-up (∼13). However, failing to relax the guess solution (hyperparameters) can result in suboptimal models when the source training set is small and very dissimilar to the target one.

Most importantly, when deployed in FFLUX simulations, TL models of atomic IQA energies and charges exhibit similar to better performance than their DL analogs. TL models behave better than DL models when it comes to guiding geometry optimization and normal mode calculations. Although the TL protocol requires the covariance matrix to fit in memory, it can be praised for being simple and intuitive, let alone that the underlying ideas can be easily coupled with existing GP scaling techniques that mitigate the huge memory requirement of full GPR model training. Work is already underway in our group to accomplish this.

## Data availability

Any data are readily available from the corresponding author upon request.

## Conflicts of interest

There are no conflicts to declare.

## Supplementary Material

CP-026-D4CP01862A-s001

## References

[cit1] Behler J., Parrinello M. (2007). Phys. Rev. Lett..

[cit2] Handley C. M., Hawe G. I., Kell D. B., Popelier P. L. (2009). Phys. Chem. Chem. Phys..

[cit3] Bartók A. P., Payne M. C., Kondor R., Csányi G. (2010). Phys. Rev. Lett..

[cit4] Larsen A. H., Mortensen J. J., Blomqvist J., Castelli I. E., Christensen R., Dułak M., Friis J., Groves M. N., Hammer B., Hargus C. (2017). J. Phys.: Condens. Matter.

[cit5] Batatia I., Kovacs D. P., Simm G., Ortner C., Csányi G. (2022). Adv. Neural Inf. Process. Syst..

[cit6] Chmiela S., Vassilev-Galindo V., Unke O. T., Kabylda A., Sauceda H. E., Tkatchenko A., Müller K.-R. (2023). Sci. Adv..

[cit7] Wang Z., Wu H., Sun L., He X., Liu Z., Shao B., Wang T., Liu T.-Y. (2023). J. Chem. Phys..

[cit8] Symons B. C. B., Bane M. K., Popelier P. L. A. (2021). J. Chem. Theory Comput..

[cit9] Symons B. C., Popelier P. L. (2022). J. Chem. Theory Comput..

[cit10] Brown M., Skelton J., Popelier P. (2023). J. Chem. Theory Comput..

[cit11] Williams C. K. and Rasmussen C. E., Gaussian processes for machine learning, MIT Press, Cambridge, MA, USA, 2006

[cit12] Snelson E., Ghahramani Z. (2006). Adv. Neural Inf. Process. Syst..

[cit13] Lázaro-Gredilla M., Quinonero-Candela J., Rasmussen C. E., Figueiras-Vidal A. R. (2010). J. Mach. Learn. Res..

[cit14] Gardner J., Pleiss G., Weinberger K. Q., Bindel D., Wilson A. G. (2018). Adv. Neural Inf. Process. Syst..

[cit15] Sun J. , Cheng L. and Miller T., Molecular Energy Learning Using Alternative Blackbox Matrix-Matrix Multiplication Algorithm for Exact Gaussian Process, 2021

[cit16] Hu C., Zeng S., Li C. (2023). Appl. Soft Comput..

[cit17] Noack M. M., Krishnan H., Risser M. D., Reyes K. G. (2023). Sci. Rep..

[cit18] Di Pasquale N. , Bane M., Davie S. J. and Popelier P. L., Wiley Online Library, 2016, pp. 2606–2616 10.1002/jcc.24486 27649926

[cit19] Burn M. J., Popelier P. L. A. (2023). Digital Discovery.

[cit20] Isamura B. K., Popelier P. L. (2023). AIP Adv..

[cit21] Noack M. M., Doerk G. S., Li R., Streit J. K., Vaia R. A., Yager K. G., Fukuto M. (2020). Sci. Rep..

[cit22] Popelier P. L. , The chemical bond II: 100 years old and getting stronger, 2016, pp. 71–117

[cit23] Burn M. J., Popelier P. L. A. (2023). J. Chem. Theory Comput..

[cit24] Brown M. L., Skelton J. M., Popelier P. L. (2023). J. Phys. Chem. A.

[cit25] van der Heide T., Kullgren J., Broqvist P., Bačić V., Frauenheim T., Aradi B. (2023). Comput. Phys. Commun..

[cit26] Klawohn S., Darby J. P., Kermode J. R., Csányi G., Caro M. A., Bartók A. P. (2023). J. Chem. Phys..

[cit27] Becke A. , The quantum theory of atoms in molecules: from solid state to DNA and drug design, John Wiley & Sons, 2007

[cit28] Blanco M., Martín Pendás A., Francisco E. (2005). J. Chem. Theory Comput..

[cit29] Guevara-Vela J. M., Francisco E., Rocha-Rinza T., Martín Pendás Á. (2020). Molecules.

[cit30] Hwang S.-G., L'Huillier B., Keeley R. E., Jee M. J., Shafieloo A. (2023). J. Cosmology Astroparticle Phys..

[cit31] Burn M. J., Popelier P. L. A. (2022). Mater. Adv..

[cit32] Pan S. J., Yang Q. (2009). IEEE Trans. Knowledge Data Eng..

[cit33] Zhuang F., Qi Z., Duan K., Xi D., Zhu Y., Zhu H., Xiong H., He Q. (2020). Proc. IEEE.

[cit34] Dral P. O. (2020). J. Phys. Chem. Lett..

[cit35] Li X., Grandvalet Y., Davoine F., Cheng J., Cui Y., Zhang H., Belongie S., Tsai Y.-H., Yang M.-H. (2020). Image Vision Comput..

[cit36] Wang J. and Chen Y., Introduction to Transfer Learning: Algorithms and Practice, Springer, 2022, pp. 275–279

[cit37] Chen M. S., Lee J., Ye H.-Z., Berkelbach T. C., Reichman D. R., Markland T. E. (2023). J. Chem. Theory Comput..

[cit38] Kamath A., Vargas-Hernández R. A., Krems R. V., Carrington T., Manzhos S. (2018). J. Chem. Phys..

[cit39] Ollar J., Mortished C., Jones R., Sienz J., Toropov V. (2017). Struct. Multidisciplinary Optimization.

[cit40] Chang Y.-C. , N-dimension golden section search: Its variants and limitations, 2009

[cit41] Yu H. and Kim S., Passive sampling for regression, 2010

[cit42] Isamura B. K., Popelier P. L. (2023). Artif. Intell. Chem..

[cit43] Mirjalili S., Mirjalili S. M., Lewis A. (2014). Adv. Eng. Software.

[cit44] Chmiela S., Sauceda H. E., Müller K.-R., Tkatchenko A. (2018). Nat. Commun..

[cit45] Bannwarth C., Ehlert S., Grimme S. (2019). J. Chem. Theory Comput..

[cit46] Kabsch W. (1976). Acta Crystallogr., Sect. A: Cryst. Phys., Diffr., Theor. Gen. Crystallogr..

[cit47] Frisch M. e , Trucks G., Schlegel H. B., Scuseria G., Robb M., Cheeseman J., Scalmani G., Barone V., Petersson G. and Nakatsuji H., Gaussian, Inc., Wallingford, CT, 2016

[cit48] Keith T. A. , TK Gristmill Software, Overland Park, KS, USA, 2019, 23

[cit49] Kandathil S. M., Fletcher T. L., Yuan Y., Knowles J., Popelier P. L. (2013). J. Comput. Chem..

[cit50] Togo A., Tanaka I. (2015). Scr. Mater..

[cit51] Guest M. F., Elena A. M., Chalk A. B. (2021). Mol. Simul..

[cit52] Todorov I. T., Smith W., Trachenko K., Dove M. T. (2006). J. Mater. Chem..

[cit53] Viering T., Loog M. (2022). IEEE Trans. Pattern Anal. Mach. Intell..

[cit54] Viering T., Mey A., Loog M. (2019). Proc. Mach. Learn. Res. vol..

[cit55] Sollich P. (2005). Lect. Notes Comput. Sci..

[cit56] Roussel O. (2011). J. Satisfiability, Boolean Modeling Comput..

[cit57] Amdahl G. , Validity of the single processor approach to achieving large scale computing capabilities, 1967

[cit58] Xu Y., Goodacre R. (2018). J. Anal. Test..

[cit59] Gallegos M., Isamura B. K., Popelier P. L., Martín Pendás A. N. (2024). J. Chem. Inf. Model..

[cit60] Santos C. F. G. D., Papa J. P. (2022). ACM Computing Surveys (CSUR).

[cit61] Mohammed R. O. and Cawley G. C., Over-fitting in model selection with Gaussian process regression, 2017

[cit62] Burn M. J., Popelier P. L. A. (2020). J. Chem. Phys..

