# Supplementary material for: Transfer learning of hyperparameters for fast construction of anisotropic GPR models: design and application to the machine-learned force field FFLUX
Source: Phys Chem Chem Phys. 2024 Sep 3;26(36):23677–91. doi: 10.1039/d4cp01862a (PMC11369757; doi:10.1039/d4cp01862a)
Supplement: CP-026-D4CP01862A-s001 [file CP-026-D4CP01862A-s001.pdf]

Supplementary Information (SI) for Physical Chemistry Chemical Physics.
This journal is © the Owner Societies 2024

Electronic Supplementary Information (ESI)

Transfer learning of hyperparameters for fast construction of anisotropic GPR models: design and application to the machine-learned force field FFLUX

Bienfait K. Isamura and Paul L.A. Popelier

Department of Chemistry, The University of Manchester, Manchester, M13 9PL, UK

Email: pla@manchester.ac.uk

Table of contents

1. **Multisource (MS) TL of hyperparameters** 2
2. **Data cleaning** 4
3. **Important control parameters for the GWO algorithm and TL protocol** 5
4. **Why choose the IHCOV approach with an anisotropic kernel?** 6
5. **Molecular learning (S-)curves of DL models** 8
6. **Training timings of DL models** 10
7. **Performance and speedup factors of baseline and FS-TL models** 11
8. **Performance and speedup factors of relaxed TL models** 12
9. **Prediction of vibrational normal modes** 13
10. **Electronic energy ranges within the set of optimization starting geometries** 17

1. Multisource (MS) TL of hyperparameters

In the main text, we have focused on the case of single-source TL of hyperparameters. The reason is that our multisource (MS) TL protocol is still under scrutiny. In this protocol, the main challenge is to decide how to transfer more than one (pseudo)-guess solution to the target model. In this section, we briefly discuss preliminary design choices aimed at allowing MS-TL of hyperparameters.

Unlike single-source TL, in multisource TL, the guessing phase returns a collection of N_s pseudo-guess solutions. In case $\zeta \neq 0$ and $N_s \leq W - 1$, all N_s pseudo-guess solutions are transferred to the target model. Also transferred is a virtual ensemble solution $\boldsymbol{\theta}_{ens}^0$ defined as shown in Eq.1,

$$\boldsymbol{\theta}_{ens}^0 = \sum_{i=1}^{N_s} \frac{(\mathcal{L}_i)^{-1}}{\mathcal{L}^{-1}} \boldsymbol{\theta}_i^0 \qquad (1)$$

where $\mathcal{L}_i$ is the optimal loss function value for the ith source model and $\mathcal{L}^{-1} = \sum_i (\mathcal{L}_i)^{-1}$. Note that the ratio ${\mathcal{L}_i}^{-1}/\mathcal{L}^{-1}$ is nothing but the weight of the ith source model in the committee of source models. Assuming that one of the source models, say model *k*, is considerably better than the others ($\mathcal{L}_k = \mathcal{L}_{best} \ll \mathcal{L}_j$ for $\forall j \neq k$) then one obtains $\theta_{ens}^0 \sim \theta_{best}^0 = \theta_k^0$.

FEREBUS users can set directives. Every configuration file contains directives and their parameters (keyword and value). In the simplest case such as .toml files, every line associates a directive with its value. For example, a line in a ferebus.toml file with the entry "full_seeding = 1" will instruct Ferebus to perform a full seeding. In particular here, FEREBUS users can set the **src_overlap** directive to 0. This choice instructs the program to generate a set of non-overlapping source datasets. Because the resulting source models are aware of a broader portion of the target domain (as compared to any unique source model), they are expected to deliver an excellent cohort of pseudo-guess solutions. Furthermore, their knowledge content is somehow condensed within the ensemble solution θ_{ens}^0, which is also transferred alongside the individual pseudo-guess solutions.

In the case $\zeta \neq 0$ and $N_s > 1$ (FS-MSTL), FEREBUS users can choose between transferring either the best among pseudo-guess solutions or the virtual ensemble solution. This is specified by setting the **fstl_msrc** directive to 0 and 1, respectively.

It is worth highlighting the similarity between our FS-MSTL approach and a recent protocol by Hu and co-workers[1]. These authors propose a GP-TL protocol that involves the training of several full GPR models (sources) on subsets of the target domain, followed by a linear projection of the guess pseudo-solutions (sets of hyperparameters) onto the target GP. Their protocol also requires

one inversion of the full covariance matrix. However, unlike our performance-based projector, their linear predictor exploits the similarity between the sources and target domains. The similarity r between two distributions A and B is defined as the inverse of the square root of their second-order Wasserstein distance $WD(A\|B)$. For a set of source solutions $\boldsymbol{\theta}^{\mathbf{0}} = [\boldsymbol{\theta}_1^0, \dots, \boldsymbol{\theta}_{N_s}^0]$, the hyperparameters of the target GPR model ($\boldsymbol{\theta}_T$) are computed using Eq.2,

$$\boldsymbol{\theta}_T = \boldsymbol{r}^T \boldsymbol{R}^{-1} \boldsymbol{\theta}^{\mathbf{0}} \qquad (2)$$

where $\boldsymbol{R}$ is an $N_s \times N_s$ similarity matrix between all pairs of source domains and $\boldsymbol{r}$ is a $N_s \times 1$ similarity matrix between the target GP and each of the sources.

Despite the soundness and elegance of this approach, it breaks down (at least in this canonical form) when there is only one source model or when the source is the same as the target. This is because $WD(S_i\|S_i) = 0$ implies that $\boldsymbol{r}^T = \infty$ and $\boldsymbol{R}$ is singular. Therefore, $\boldsymbol{\theta}_T$ is undefined.

As mentioned before, we are still carrying out systematical tests of the MS-TL protocol. However, based on the results reported in Section 4.2 of the main text, we can confidently say that single-sourcing already works perfectly and we are willing to see how multi-sourcing affects the quality of target models.

2. Data cleaning

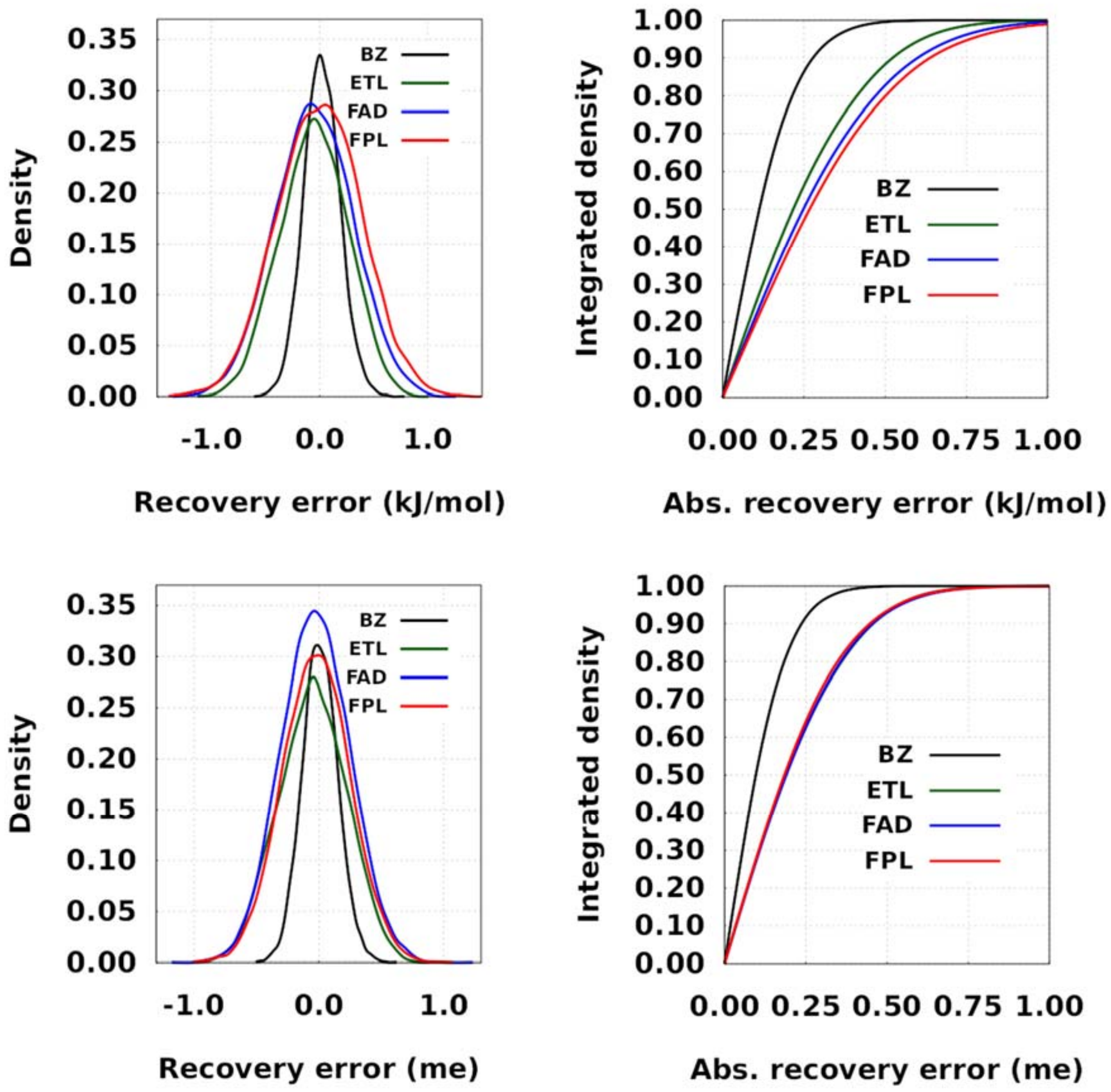

Figure S1. Kernel density fitting of the recovery/reconstruction errors (IQA and Q00) associated with each molecule in our datasets. The left plots show the distribution of recovery errors in the initial dataset of 10,000 geometries, while the right plots indicate the proportion of geometries whose recovery error is lower or equal to some value. The latter curves are respectively truncated at thresholds of 1 kJ/mol and 1 me for E_{IQA} and Q_{00} since these thresholds accommodate more than 99% of the geometries. Of the initial 10,000 geometries, the cleaning process removed 0, 6, 40, and 94 geometries of BZ, ETL, FAD, and FPL, respectively. This very low removal rate testifies to the high quality of our training data whose noise level lies within the limit of chemical accuracy (4.2 kJ/mol). In particular, the BZ data are very clean, with reconstruction errors lower than 0.5 kJ/mol and 0.5 me for the majority of geometries (> 99%).

3. Important control parameters for the GWO algorithm and TL protocol

Table S1. Important control parameters for the grey wolf optimizer and the TL protocol. Default values are also provided as defined in the config.f90 module of FEREBUS.

Parameter	Description	Default value
r_{max}	Controls the extent of perturbation of the guess solution in TL jobs.	0.25
a_{max}	The maximum value of the a(t) parameter of the grey wolf optimiser intended to maintain the balance between exploration and exploitation of the hyperparameter space.	2.0
**number_of_agents**	Number of active agents or candidate solutions used to scrutinise the hyperparameter space.	50
τ	Maximum number of training iterations. In the FEREBUS configuration file, it is referred to as "max_iterations".	200
χ	The number of relaxation steps. It is defined as $\chi = \zeta \times \tau$, where ζ is the relaxation weight and τ is the maximum number of iterations.	20
**full_seeding**	Specifies whether the seeding phase should be extended over τ steps, or $\tau - \chi$ steps. Like other flags, it takes two possible values, 0 (disabled) or 1 (enabled).	1
**n**	Number of lucky agents to be promoted when updating the positions of non-leader solutions.	5
**p**	The frequency at which lucky agents are promoted towards the centroid of the leaders' positions.	5
**r**	Defines the extent of random walks of lucky agents around the centroid of leaders.	0.20

4. Why choose the IHCOV approach with an anisotropic kernel?

The hyperparameters of a GPR model can be optimized by maximizing the log marginal likelihood function of the GP. This popular approach, known as Type-II maximum likelihood (ML-II), offers the advantage of being analytically tractable[2]. As a result, any off-the-shelf gradient-based optimizer can be used to scrutinize the hyperparameter space (HS).

Despite its practical suitability, the ML-II protocol suffers from well-known limitations: it is very sensitive to the presence of outliers in the training data[3] and tends to be less robust to model misspecification as compared to cross-validation (CV)[4]. Moreover, the ML-II protocol is prone to numerical instability in the noise-free regime (i.e., when $\sigma_n^2 = 0$) where errors are accentuated by vector-matrix-vector operations involving a less regularised and ill-conditioned covariance matrix[5]. Fortunately, FEREBUS is now equipped with an alternative cross-validation (CV) protocol for hyperparameter optimization, more resistant to model misspecification and less costly than the popular leave-one-out CV (see main text for more details).

In this section, we justify the choice put on the IHOCV protocol over ML-II and anisotropic kernels over their isotropic analogs when it came to designing our TL protocol. We compared the performance of DL models trained on 8000 geometries (and tested on the same 1000 geometries) using isotropic and anisotropic kernels in the framework of the IHOCV and ML-II protocols. The isotropic IHOCV-GPR models were trained *via* a grid-based search along the directions of the unique kernel parameter θ and the regularisation noise σ_n^2. The grid was made of 50×50 points, or 50 points in each dimension of the HS. The latter space was defined as $[0.0, 3.0]$ and $[10^{-14}, 10^{-4}]$ for θ and σ_n^2, respectively. The nodes/points along the second dimension were positioned on the logarithmic scale by uniformly sampling (on that scale) 50 points between the lower and upper boundaries.

Table S1 reports the results obtained in terms of the reconstruction of the molecular IQA energy and charge. The data in this Table demonstrate the consistent superiority of IHOCV^{aniso} models over IHOCV^{iso} and ML-II^{aniso} models when it comes to reproducing IQA energies and charges. IHOCV^{iso} models compete with IHOCV^{aniso} in the case of IQA energies of BZ, while ML-II^{aniso} models generally exhibit the worst generalization capability, sometimes leading to MAEs being almost twice as large as those of IHOCV^{aniso} models. These findings motivated our choice for the IHOCV model selection approach with an anisotropic kernel.

Table S2. Performance (MAEs) of DL models trained on 8000 geometries using both an isotropic and an anisotropic kernel within the IHCOV framework. Anisotropic ML-II models are also reported for comparison.

Model	BZ	ETL	FAD	FPL
		**IQA energy (kJ/mol)**		
$IHOCV^{aniso}$	0.210	0.977	0.489	0.604
$IHOCV^{iso}$	0.213	1.409	0.561	0.729
$ML\text{-}II^{aniso}$	0.349	1.804	0.858	0.821
		**Q_{00} (me)**		
$IHOCV^{aniso}$	0.274	1.954	0.423	0.903
$IHOCV^{iso}$	0.377	3.198	0.511	1.203
$ML\text{-}II^{aniso}$	0.398	2.351	0.659	1.113

5. Molecular learning (S-)curves of DL models

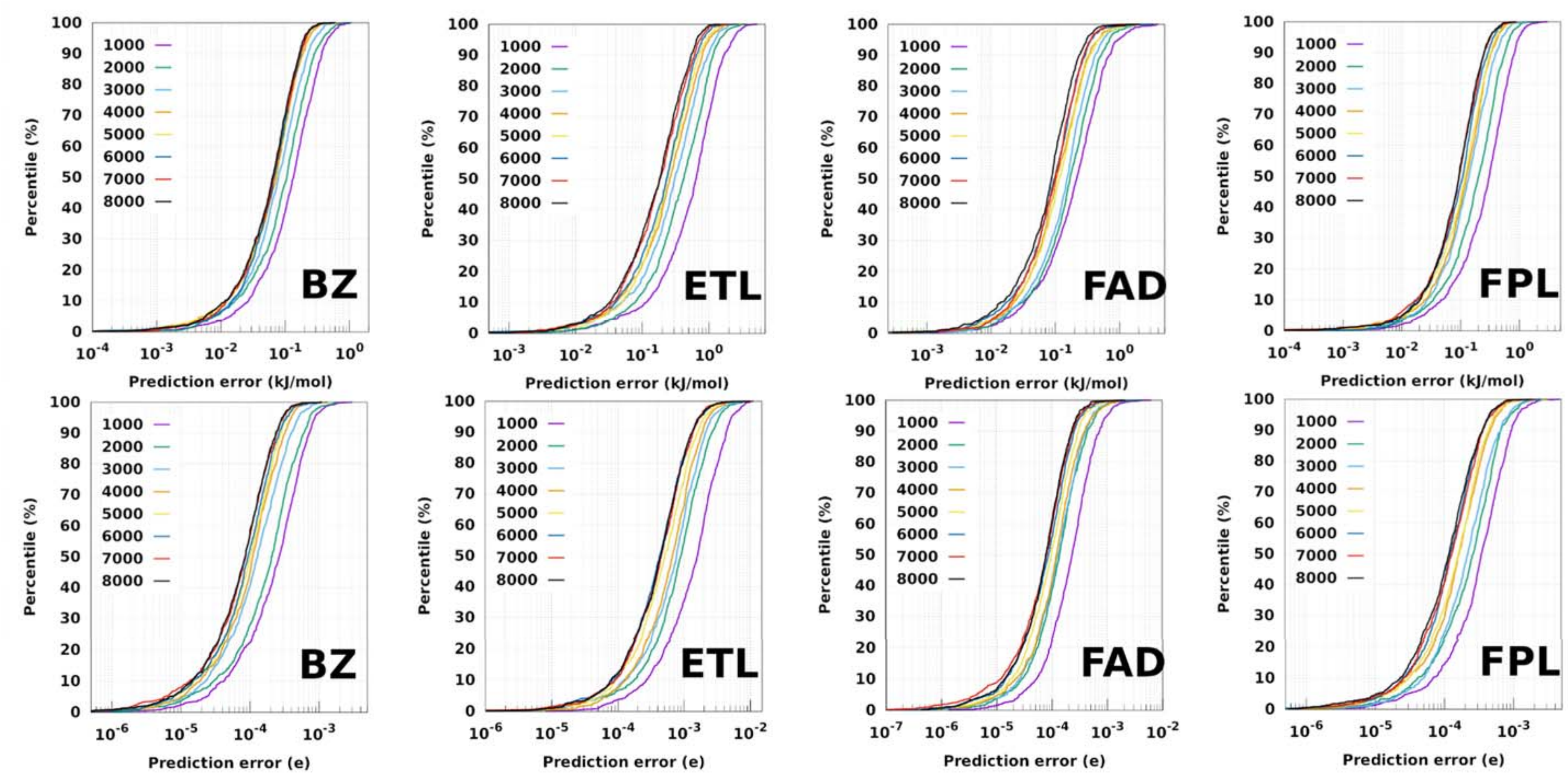

Figure S2. Molecular learning S-curves of DL E_{IQA} and Q_{00} models. These curves move to the left as the model is improved through data augmentation. As expected, the purple curve at the rightmost corresponds to the smallest training set, while the black curve is at the leftmost.

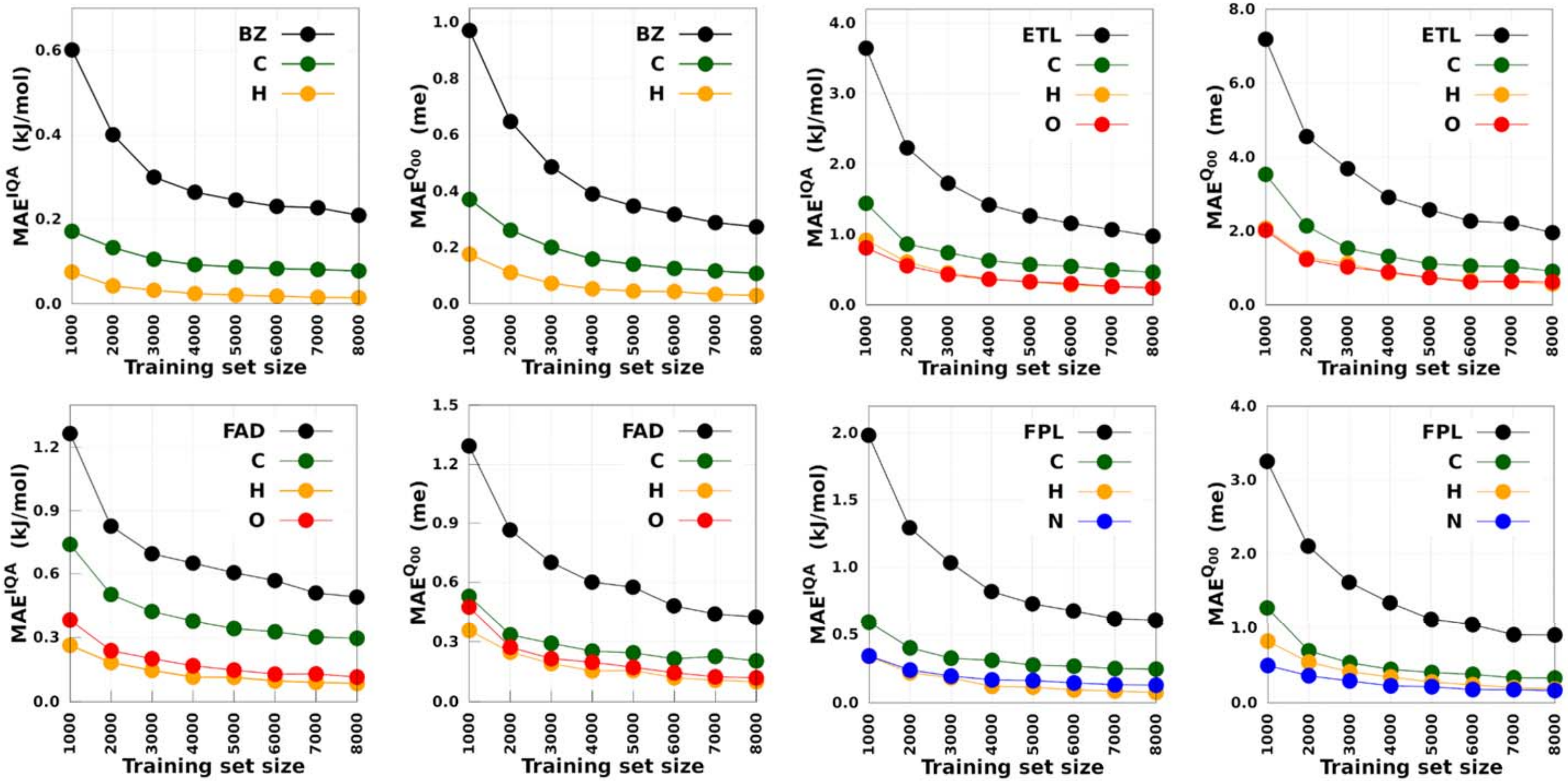

Figure S3. Learning curves of anisotropic GPR models trained on 1000 to 8000 geometries. Element-wise models (collections of atomic models of the same element) saturate faster than molecular ones.

6. Training timings of DL models

Table S3. Training timings of atomic GPR models of BZ, ETL, FAD and FPL. The wall times are shown in parentheses. All timings are expressed in hours and correspond to the short CPU/core or wall time recorded for a set of models trained on the same number of geometries.

N	BZ	ETL	FAD	FPL
1000	0.203 (0.240)	0.163 (0.189)	0.176 (0.207)	0.222 (0.266)
2000	0.516 (0.598)	0.409 (0.467)	0.455 (0.521)	0.559 (0.635)
3000	0.996 (1.138)	0.843 (0.968)	0.828 (0.940)	0.999 (1.150)
4000	1.703 (1.868)	1.242 (1.412)	1.346 (1.457)	1.713 (1.937)
5000	2.452 (2.689)	1.763 (1.938)	2.072 (2.333)	2.539 (2.783)
6000	3.112 (3.486)	2.732 (3.075)	3.234 (3.569)	3.371 (3.716)
7000	4.176 (4.601)	3.465 (3.813)	4.245 (4.615)	4.732 (5.118)
8000	5.751 (6.675)	4.990 (5.508)	6.033 (6.488)	6.498 (7.101)

7. Performance and speedup factors of baseline and FS-TL models

Table S4. Predictive MAEs of baseline and FS-TL models for the reconstruction of molecular energies (in kJ/mol) and charges (in me). DL data are provided for comparison.

Model	BZ	ETL	FAD	FPL
		**IQA energy (kJ/mol)**		
DL	0.210	0.977	0.489	0.604
$DL^{1\%}$	7.509	16.022	9.770	11.725
$DL^{10\%}$	0.718	4.236	1.743	2.881
$DL^{25\%}$	0.400	2.231	0.826	1.294
$TL_{0.01}^{0.00}$	0.391	4.001	2.107	1.839
$TL_{0.10}^{0.00}$	0.338	2.202	1.068	1.164
$TL_{0.25}^{0.00}$	0.215	1.273	0.542	0.615
RBL	0.376	3.988	1.293	1.868
		**Q_{00} (me)**		
DL	0.274	1.954	0.423	0.903
$DL^{1\%}$	10.686	29.873	11.562	23.339
$DL^{10\%}$	1.132	8.378	2.842	3.637
$DL^{25\%}$	0.647	4.552	0.866	2.103
$TL_{0.01}^{0.00}$	0.542	9.496	1.626	3.221
$TL_{0.10}^{0.00}$	0.389	3.236	1.217	1.831
$TL_{0.25}^{0.00}$	0.275	2.255	0.591	1.035
RBL	0.534	6.269	1.369	2.834

Table S5. Speedup factors of baseline and FS-TL models as compared to the largest DL models. These metrics were computed as the ratio between the wall time of the test model and that of the reference DL model.

Model	BZ	ETL	FAD	FPL
DL	1.0	1.0	1.0	1.0
$DL^{1\%}$	513.5	612.0	648.8	591.8
$DL^{10\%}$	35.7	39.1	38.2	35.2
$DL^{25\%}$	11.2	11.8	12.5	11.2
$TL_{0.01}^{0.00}$	109.4	117.2	115.9	109.2
$TL_{0.10}^{0.00}$	39.0	34.9	41.9	35.5
$TL_{0.25}^{0.00}$	13.7	12.7	13.3	13.3
RBL	20.4	17.3	17.8	19.2

8. Performance and speedup factors of relaxed TL models

Table S6. Predictive MAEs of the shortly relaxed TL models for the reconstruction of molecular energies (in kJ/mol) and charges (in me). DL data are provided for comparison.

Model	BZ	ETL	FAD	FPL
		**IQA energy (kJ/mol)**		
DL	0.210	0.977	0.489	0.604
$TL_{0.01}^{0.05}$	0.268	1.659	0.859	1.102
$TL_{0.10}^{0.05}$	0.261	1.302	0.648	1.045
$TL_{0.25}^{0.05}$	0.207	1.031	0.482	0.626
$TL_{0.01}^{0.10}$	0.262	1.332	0.782	0.888
$TL_{0.10}^{0.10}$	0.232	1.307	0.554	0.829
$TL_{0.25}^{0.00}$	0.215	0.910	0.462	0.597
		**Q_{00} (me)**		
DL	0.274	1.954	0.423	0.903
$TL_{0.01}^{0.05}$	0.364	3.279	0.723	1.603
$TL_{0.10}^{0.05}$	0.324	2.812	0.709	1.597
$TL_{0.25}^{0.05}$	0.270	2.072	0.415	0.978
$TL_{0.01}^{0.10}$	0.340	2.945	0.547	1.437
$TL_{0.10}^{0.10}$	0.303	2.249	0.538	1.371
$TL_{0.25}^{0.00}$	0.298	1.881	0.418	0.867

Table S7. Speedup factors of the shortly relaxed TL models in comparison with the largest DL models. These metrics were computed as the ratio between the wall time of the test model and that of the reference DL model.

Model	BZ	ETL	FAD	FPL
DL	1.0	1.0	1.0	1.0
$TL_{0.01}^{0.05}$	16.8	22.2	19.7	17.2
$TL_{0.10}^{0.05}$	14.1	15.7	16.3	14.0
$TL_{0.25}^{0.05}$	8.0	8.1	8.3	7.9
$TL_{0.01}^{0.10}$	8.6	10.9	11.7	9.1
$TL_{0.10}^{0.10}$	8.7	8.5	9.4	9.5
$TL_{0.25}^{0.00}$	5.3	6.0	6.1	5.7

9. Prediction of vibrational normal modes

Table S8. Reference (GAUSSIAN) and FFLUX-predicted vibrational frequencies of BZ using both DL and TL models. The absolute deviations (Δ) of each model from the reference are provided. All the frequencies are expressed in cm^{-1}. The worst predicted mode is written in bold.

Mode	Ref.	DL	TL	Δ^{DL}	Δ^{TL}	Mode	Ref.	DL	TL	Δ^{DL}	Δ^{TL}
**1**	412.52	418.91	419.93	6.39	7.41	**16**	1173.23	1184.45	1184.20	11.23	10.97
**2**	412.56	422.80	424.41	10.24	11.85	**17**	1197.60	1208.04	1207.57	10.44	9.97
**3**	623.14	629.89	630.79	6.75	7.65	**18**	1197.70	1208.61	1209.23	10.90	11.52
**4**	623.16	632.00	634.28	8.84	11.12	**19**	1334.18	1349.99	1352.92	15.81	18.74
**5**	693.17	706.62	707.00	13.45	13.83	**20**	1387.91	1390.81	1390.91	2.90	3.00
**6**	721.40	726.41	728.54	5.01	7.14	**21**	1515.68	1518.29	1516.42	2.61	0.73
**7**	870.75	879.82	880.75	9.06	10.00	**22**	1515.78	1521.59	1522.68	5.81	6.90
**8**	870.87	881.51	884.29	10.64	13.42	**23**	1633.46	1640.24	1643.15	6.78	9.69
**9**	996.39	1002.24	1003.69	5.85	7.30	**24**	1633.71	1648.62	1650.31	14.90	16.59
**10**	996.50	1004.88	1006.15	8.38	9.65	**25**	3156.79	3191.16	3188.43	34.37	31.65
**11**	1013.78	1027.89	1028.75	14.11	14.97	**26**	3166.50	3206.08	3199.60	39.58	33.10
**12**	1022.99	1033.03	1032.76	10.04	9.77	**27**	3166.73	3207.18	3209.83	40.45	43.10
**13**	1030.52	1034.46	1035.11	3.94	4.59	**28**	3181.90	3218.44	3220.50	36.53	38.60
**14**	1060.45	1068.52	1070.80	8.07	10.35	**29**	3182.13	3221.36	3223.33	39.23	41.20
**15**	1060.50	1075.26	1076.82	14.76	16.32	**30**	**3191.96**	**3241.21**	**3246.21**	**49.26**	**54.25**

Table S9. Reference (GAUSSIAN) and FFLUX-predicted vibrational frequencies of ETL using both DL and TL models. The absolute deviations (Δ) of each model from the reference are provided. All the frequencies are expressed in cm^{-1}. The worst predicted mode is written in bold.

Mode	Ref.	DL	TL	Δ^{DL}	Δ^{TL}	Mode	Ref.	DL	TL	Δ^{DL}	Δ^{TL}
**1**	**256.38**	**179.70**	**193.16**	**76.67**	**63.22**	**12**	1416.25	1403.48	1402.09	12.77	14.16
**2**	269.31	263.64	270.61	5.66	1.31	**13**	1488.27	1491.66	1484.53	3.39	3.74
**3**	420.17	426.61	424.75	6.44	4.58	**14**	1493.21	1500.76	1496.16	7.55	2.95
**4**	805.89	809.82	800.21	3.93	5.68	**15**	1517.07	1532.00	1525.22	14.93	8.15
**5**	881.52	896.25	891.94	14.73	10.42	**16**	2989.56	3016.22	2997.57	26.66	7.97
**6**	1057.07	1063.59	1054.57	6.52	2.50	**17**	3019.32	3047.34	3030.00	28.02	10.68
**7**	1067.13	1088.80	1087.07	21.67	19.93	**18**	3063.81	3109.56	3088.69	45.76	24.88
**8**	1131.61	1144.95	1133.66	13.34	2.05	**19**	3084.29	3147.51	3135.61	63.22	51.32
**9**	1279.45	1281.23	1267.93	1.79	11.52	**20**	3098.39	3154.27	3151.34	55.88	52.95
**10**	1368.16	1361.02	1347.71	7.14	20.45	**21**	3809.63	3746.97	3746.66	62.66	62.96
**11**	1403.12	1393.62	1385.11	9.50	18.01						

Table S10. Reference (GAUSSIAN) and FFLUX-predicted vibrational frequencies of FAD using both DL and TL models. The absolute deviations (Δ) of each model from the reference are provided. All the frequencies are expressed in cm^{-1}. The worst predicted mode is written in bold.

Mode	Ref.	DL	TL	Δ^{DL}	Δ^{TL}	Mode	Ref.	DL	TL	Δ^{DL}	Δ^{TL}
**1**	78.50	72.84	72.78	5.65	5.72	**13**	1265.35	1257.45	1260.43	7.91	4.92
**2**	170.22	165.36	169.58	4.86	0.65	**14**	1267.09	1285.62	1280.66	18.53	13.57
**3**	185.46	173.27	172.81	12.19	12.65	**15**	1398.36	1396.06	1392.98	2.30	5.38
**4**	201.45	209.26	207.55	7.80	6.10	**16**	1403.70	1407.64	1403.33	3.94	0.37
**5**	261.46	252.20	249.89	9.25	11.57	**17**	1441.56	1447.64	1442.11	6.09	0.56
**6**	267.94	271.07	269.54	3.13	1.59	**18**	1467.60	1490.09	1483.71	22.49	16.11
**7**	681.93	689.33	690.22	7.40	8.29	**19**	1704.04	1704.15	1697.86	0.11	6.18
**8**	714.75	719.49	718.94	4.74	4.18	**20**	1777.50	1780.18	1776.14	2.67	1.37
**9**	982.05	944.30	950.11	37.75	31.94	**21**	3063.01	3089.31	3085.91	26.30	22.90
**10**	1001.00	978.47	977.41	22.54	23.59	**22**	3095.92	3128.52	3091.16	32.60	4.76
**11**	1073.68	1048.82	1047.69	24.86	25.99	**23**	**3107.15**	**3162.07**	**3149.80**	**54.92**	**42.65**
**12**	1101.08	1078.92	1078.48	22.15	22.60	**24**	3183.10	3204.25	3195.49	21.16	12.39

Table S11. Reference (GAUSSIAN) and FFLUX-predicted vibrational frequencies of FPL using both DL and TL models. The absolute deviations (Δ) of each model from the reference are provided. All the frequencies are expressed in cm^{-1}. The worst predicted mode is written in bold.

Mode	Ref.	DL	TL	Δ^{DL}	Δ^{TL}	Mode	Ref.	DL	TL	Δ^{DL}	Δ^{TL}
**1**	71.35	37.75	38.22	33.60	33.13	**16**	1243.14	1273.46	1263.87	30.32	20.74
**2**	267.35	283.63	281.26	16.28	13.90	**17**	1316.44	1329.02	1324.72	12.58	8.28
**3**	321.31	329.70	327.20	8.39	5.89	**18**	1385.67	1388.73	1387.41	3.06	1.74
**4**	501.10	523.81	522.06	22.70	20.95	**19**	1415.49	1431.22	1427.45	15.73	11.97
**5**	634.52	657.01	652.88	22.49	18.36	**20**	1429.95	1449.51	1447.69	19.56	17.74
**6**	652.58	673.89	673.75	21.31	21.17	**21**	1490.85	1490.61	1485.20	0.23	5.65
**7**	687.24	704.87	702.36	17.63	15.12	**22**	1493.69	1507.73	1496.88	14.04	3.19
**8**	787.06	803.86	797.77	16.81	10.71	**23**	1509.49	1521.80	1526.13	12.31	16.63
**9**	869.18	883.21	877.03	14.04	7.85	**24**	1620.10	1629.71	1621.39	9.61	1.29
**10**	947.48	965.38	961.84	17.90	14.36	**25**	**3034.72**	**3127.21**	3107.33	**92.49**	72.61
**11**	1000.55	1007.85	1002.73	7.30	2.19	**26**	3087.44	3145.26	3142.42	57.82	54.97
**12**	1011.55	1030.07	1022.99	18.51	11.44	**27**	3111.86	3174.60	3171.28	62.74	59.42
**13**	1066.40	1077.13	1071.24	10.73	4.84	**28**	3245.60	3296.77	3293.02	51.17	47.42
**14**	1067.68	1083.64	1075.31	15.96	7.63	**29**	**3264.28**	3329.79	**3339.52**	65.51	**75.24**
**15**	1164.37	1180.46	1182.15	16.09	17.78	**30**	3681.75	3694.84	3696.64	13.08	14.89

10. Electronic energy ranges within the set of optimization starting geometries

Table S12. Range of electronic energies (in kJ/mol) within the pool of starting geometries for FFLUX optimization. These electronic energies were computed at the reference level of theory, i.e., B3LYP/aug-cc-pVTZ for BZ and ETL, and B3LYP/6-31+G(d,p) for FAD and FPL. For comparison, we also provide similar ranges within the training sets (TS). DF is the displacement factor used in eq (31) of the main text, which acts as a scaling factor.

DF	Electronic energy range (kJ/mol)			
	**BZ**	**ETL**	**FAD**	**FPL**
**0.1**	25.2	21.6	44.0	30.8
**0.2**	104.9	100.3	183.6	165.8
**0.3**	246.9	267.6	442.2	503.7
**0.4**	462.2	578.0	863.7	1242.9
**0.5**	765.8	1127.1	1520.0	1823.3
**TS**	88.5	154.6	102.4	92.9

References

1. C. Hu, S. Zeng and C. Li, *Applied Soft Computing*, 2023, **148**, 110866.
2. C. K. Williams and C. E. Rasmussen, *Gaussian processes for machine learning*, MIT press, Cambridge, MA, USA, 2006.
3. P. Jylänki, J. Vanhatalo and A. Vehtari, *J. of Machine Learning Research*, 2011, **12**, 3227-3257.
4. F. Bachoc, *Computational Statistics & Data Analysis*, 2013, **66**, 55-69.
5. B. K. Isamura and P. L. A. Popelier, *AIP Advances*, 2023, **13**, 095202.
